# Multistability in Large Scale Models of Brain Activity

**DOI:** 10.1371/journal.pcbi.1004644

**Published:** 2015-12-28

**Authors:** Mathieu Golos, Viktor Jirsa, Emmanuel Daucé

**Affiliations:** 1 Aix-Marseille Université, Inserm, INS UMR_S 1106, Marseille, France; 2 Ecole Centrale Marseille, Marseille, France; University of Toronto, CANADA

## Abstract

Noise driven exploration of a brain network’s dynamic repertoire has been hypothesized to be causally involved in cognitive function, aging and neurodegeneration. The dynamic repertoire crucially depends on the network’s capacity to store patterns, as well as their stability. Here we systematically explore the capacity of networks derived from human connectomes to store attractor states, as well as various network mechanisms to control the brain’s dynamic repertoire. Using a deterministic graded response Hopfield model with connectome-based interactions, we reconstruct the system’s attractor space through a uniform sampling of the initial conditions. Large fixed-point attractor sets are obtained in the low temperature condition, with a bigger number of attractors than ever reported so far. Different variants of the initial model, including (i) a uniform activation threshold or (ii) a global negative feedback, produce a similarly robust multistability in a limited parameter range. A numerical analysis of the distribution of the attractors identifies spatially-segregated components, with a centro-medial core and several well-delineated regional patches. Those different modes share similarity with the fMRI independent components observed in the “resting state” condition. We demonstrate non-stationary behavior in noise-driven generalizations of the models, with different meta-stable attractors visited along the same time course. Only the model with a global dynamic density control is found to display robust and long-lasting non-stationarity with no tendency toward either overactivity or extinction. The best fit with empirical signals is observed at the edge of multistability, a parameter region that also corresponds to the highest entropy of the attractors.

## Author Summary

Recent developments in non-invasive brain imaging allow reconstructing axonal tracts in the human brain and building realistic network models of the human brain. These models resemble brain systems in their network character and allow deciphering how different regions share signals and process information. Inspired by the metastable dynamics of the spin glass model in statistical physics, we systematically explore the brain network’s capacity to process information and investigate novel avenues how to enhance it. In particular, we study how the brain activates and switches between different functional networks across time. Such non-stationary behavior has been observed in human brain imaging data and hypothesized to be linked to information processsing. To shed light on the conditions under which large-scale brain network models exhibit such dynamics, we characterize the principal network patterns and confront them with modular structures observed both in graph theoretical analysis and resting-state functional Magnetic Resonance Imaging (rs-fMRI).

## Introduction

The brain’s resting state activity shows large-scale fluctuating spatiotemporal patterns as observed in neuroelectric, neuromagnetic and hemodynamic brain imaging. Biswal and colleagues [1] demonstrated in their seminal work that co-activated brain regions maintain a high correlation of BOLD (blood oxygen level dependent) signal fluctuations at rest, identifying a resting-state network of functionally connected regions. Interestingly, these patterns show intermittent co-activations of more or less distant brain regions, which are known from task conditions. Deciphering the logic behind this organized activity is the subject of intense investigation. The observation that there are relatively consistent distributed patterns of activity during rest suggests that it might be possible to characterize network dynamics through a low-dimensional set of Resting State Network (RSN) patterns [2–11], around which the dynamics is organized. Furthermore, recent experimental functional Magnetic Resonance Imaging (fMRI) studies demonstrated that the resting state dynamics is not stationary [12] in the sense that the set of functional correlations between brain areas, the so-called Functional Connectivity (FC), changes on a time scale of tens of seconds to minutes. Although non-stationarity is not in conflict with a spatiotemporal organization around a low-dimensional set of RSN patterns, it certainly renders its interpretation more difficult. The non-stationary brain network dynamics was named Functional Connectivity Dynamics (FCD) [13] and shown to be also present in computational network models. These models are typically based on neural population models of the Wilson-Cowan type, which are coupled by a biologically realistic human connectivity matrix, the so-called Connectome, derived from diffusion weighted tensor imaging (DTI). A necessary condition for the emergence of non-stationary FCD is a sufficiently strong nonlinearity in the network population models [13], which then enables the network dynamics to generate brain activation states that cannot be linked trivially to its structural connectivity (SC). These brain activation states are thus true consequences of the mutual presence of network connectivity and nonlinear dynamic interactions across network nodes. Ghosh et al [14] referred to the noise-driven stochastic process of transient activations of brain states as the exploration of the brain’s dynamic repertoire, emphasizing the potential functional relevance of these states. The number of states has been previously reported to be low (5 through 10), but neither systematic analysis of its number and character, nor an empirical validation has been performed so far.

Here we systematically analyze the capacity of a connectome-based network to store network patterns. The storage is accomplished through attractors, which represent regimes in state space that attract system trajectories as the network evolves in time. If the attracting regime is a point, we name it a fixed-point attractor. Our key hypothesis is that the capacity to generate transient fluctuating patterns over time stems from its ability to create a large number of multistable fixed point attractors. The set of attractors is directly linked to the observable variability in the fluctuating network signal in the presence of noise. Brain signal variability at rest has been proposed to be a good biomarker for various brain diseases, but in particular highlighted in studies of the aging brain [15–17]. For these reasons a systematic characterization of the range of attractors is critical and timely.

To perform such systematic pattern identification and characterization, we adapt a deterministic variant of the spin-glass dynamics, called the Hopfield “graded neuronal response” model [18]. Related models such as the Brunel-Wang and Wong-Wang system have been used previously in connectome-based modeling [14, 19, 20] and are mostly constrained to multi-stable fixed-point dynamics and threshold behavior for an isolated network node. When connected in a network, these behaviors are changed and novel network states may emerge [13]. Conversely, the Hopfield network model has the advantage that some important dynamic quantifiers, especially relevant to signal complexity, can be computed analytically. More sophisticated neural population models have been developed showing complex oscillatory behaviors [21–25] and have been successfully applied to the exploration of mostly encephalographic large-scale network dynamics [26–29]. On these large spatial scales, time delays have been demonstrated to be critical for the emergent network oscillations [26, 30], which are known to be non-trivial to simulate computationally. Recent neuroinformatics platforms such as The Virtual Brain [31–34] aid in these efforts with the goal to enable the fusion of structural and functional empirical data for large-scale modeling purposes.

However, modern computational neuroscience has also demonstrated the importance of a variety of mechanisms for neural network functioning beyond traditional excitatory and inhibitory coupling, as for instance diffusion of ions in the interstitial space or glial activity and astrocytes [35]. Such microscopic processes are modeled either with detailed biophysical models dependent on the neuronal membrane voltage dynamics, buffering by glial cells, and diffusion to the blood vessels or by more abstract models of so-called activator-inhibitor type. Our approach, however, limits the degrees of freedom and parameters of the Hopfield model. To absorb unconventional coupling mechanisms at least to some degrees, we generalize the traditional Hopfield model to include a total of three dynamic variants comprising dynamic threshold adaptation and mimicking effects of excitability changes and depolarization block [36, 37].

Using a fine-grained connectome (composed of up to one thousand nodes) we construct three network models and systematically explore the range of their dynamic repertoires via attractor counts and bifurcation analyses. Then we characterize these dynamic repertoires functionally and validate them against empirical fMRI data. Finally, we scrutinize the degree of non-stationarity in each of the network models.

## Results

### Defining the dynamic repertoire: The attractor landscape

**Fig 1:**
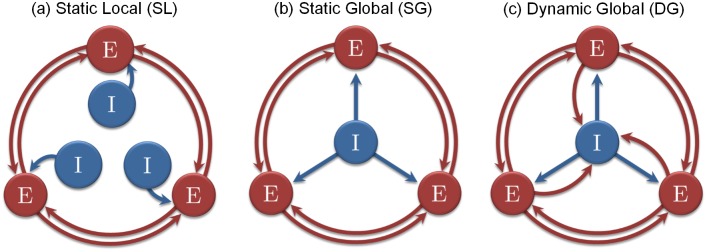
The different schemes used in simulations. Each excitatory node (E) corresponds to one anatomical region of interest (with 998 nodes simulated in most of our numerical investigations). The inhibition (I) takes the form of a linear threshold in our model. It can be local (L) or global (G), and static (S) or dynamic (D).

Here we systematically explore the capacity to store fixed-point attractors in connectome-based Hopfield networks. We consider three variants of the graded-response Hopfield model (see Fig 1). The different models are described in Eqs (1), (2) and (3)–(5) and are referred to as the “Static and Local” (SL) threshold model (equivalent to the traditional Hopfield network node), the “Static and Global” (SG) threshold model, and the “Dynamic and Global” (DG) threshold model. The two latter are variants of the first model (SL) testing different interplays between the excitatory and inhibitory influences.

Two principal control parameters are considered hereafter: the gain *G*, representing the node excitability, and the scaling factor *P*, representing the ratio of excitation over inhibition. We initially present a detailed investigation of the SL model, its multistable behavior and bifurcations. We then extend the attractor analysis to all three models providing a detailed account of dependence of the attractor landscape on the gain *G* and the scaling factor *P* in the high-gain condition.

#### Multistability and bifurcations of the SL network model

The concept of attractor is central to this study. It stems from the theory of dynamical systems. An attractor is a sub-region of the state space where a trajectory tends to converge. The convergence toward an attractor is called the “relaxation” of the dynamics. In the case of a fixed-point attractor, the final activity remains stationary over time after reaching its attractor. A single system can have one or multiple attractors. The case where more than one attractor is present is called the “multistable” case.

In the multistable case, the state space can be split in several regions, each of them driving the dynamics toward a different attractor. Those regions are called the “attractor basins”. High-dimensional systems can have very complex flows with many attractor basins. In that case, it is often impossible to deduce the attractor basins from the system description solely. Simulations must be performed to uncover the behavior of the system. A sampling of the space of initial conditions (see Material and Methods) can help to figure out the shape of those attractor basins. Then, the width and extent of the estimated attractor basins provides a reliable estimate of the stability of the corresponding attractors in the noise condition.

**Fig 2:**
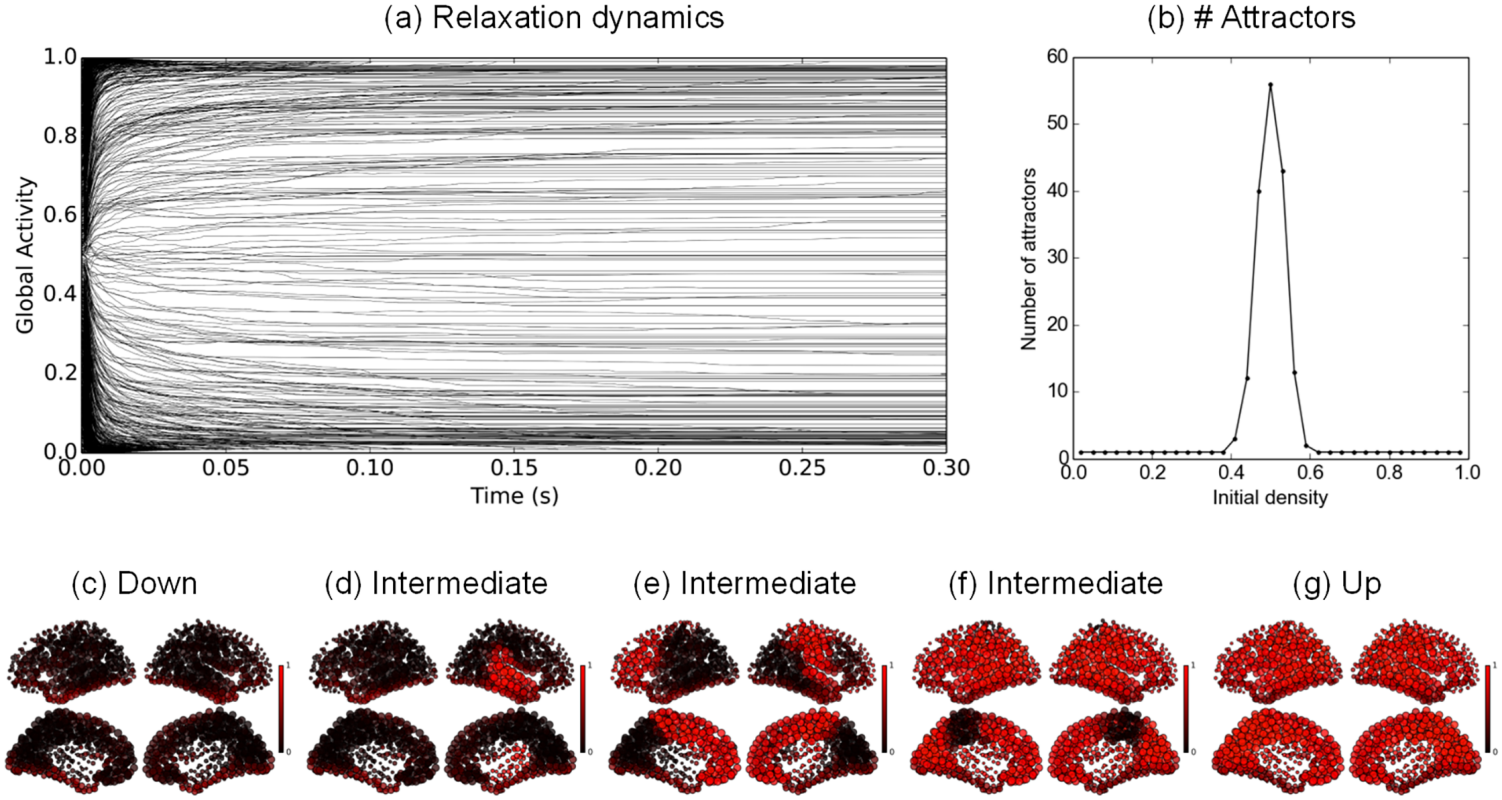
Hopfield graded-response connectome dynamics. **a**: Time course of the average network activity after 1,000 different random initializations. **b**: Distribution of the number of attractors depending on the initial density activity. **c–g**: Example final activation patterns: **c** is obtained for an initial density *f*_0_ = 0.02, **d–f** for *f*_0_ = 0.5 and **g** for *f*_0_ = 0.98. *P* = 1, *G* = 900.

The SL network comprises one thousand nodes, each node representing a parcel according to the brain parcellation proposed in [38] (see the Material and Methods section). Fig 2 gives the relaxation dynamics obtained with initial conditions varying from very sparse to very dense. 100 trajectories were simulated for an initial activity varying from 2% to 98% average activation (with 3% steps), making a total of 3,300 simulations. The time course of the average activation provides a synthetic view of the relaxation dynamics toward the final attractors.

We plot a sample of such average trajectories in Fig 2a, with each gray line representing a different average time course from a different initial condition. The convergence toward a fixed point is rather fast, with most of the final attractors attained after approximately 100 ms. The final average activity seems to be widespread across the [0, 1] interval. When close to 0, the corresponding pattern of activity is expected to have few active nodes. Conversely, when close to 1, the corresponding pattern of activity is expected to have most of its nodes active. From a finer visual inspection, the final distribution of the average activities is not uniform, with a majority of trajectories converging either to the highest level of activity, or to the lowest one. These two final average states correspond to the two dominant attractors of the dynamics, i.e. the high activity state (“Up” state) and the low activity state (“Down” state).

For each initial density, a soft clustering was applied in order to provide a rough estimate of the number of final attractors (see the Material and Methods section). The results are presented in Fig 2b. The peak of pattern variability is observed for an initial density of 0.5 (50% nodes activation), with 56 different final attractors obtained for 100 different random initializations. For initial densities lower than 0.5, the dynamics almost surely converges to the “Down” state. Conversely, with initial densities higher than 0.5, it converges to the “Up” state.

The spatial spread of the final attractors can be visualized on the cortical surface. Fig 2c represents the “Down” state (inactive state). Fig 2g represents the “Up” state. Fig 2d–2f displays intermediary states with contrasted activities and a clear separation between excited nodes and depressed nodes. The non-trivial attractors spatially vary with densities from very sparse to very dense. The general trends are (i) a shared activity on neighbour nodes, reflecting the dominant local connections, (ii) a partial left-right symmetry, with contra-lateral regions often co-active and (iii) a minority of nodes disconnected from the rest of the network, displaying a central 0.5 activity level, independent of the other nodes (around the entorhinal cortex, parahippocampal cortex and fusiform gyrus). The final patterns are rather “patchy”, with full regions being arbitrarily on or off from attractor to attractor. The peak of multistability is observed for initial patterns of density close to 0.5, while the trivial “up” (resp. “down”) attractors are attained for denser (resp. sparser) patterns of initial conditions. Thus the dynamics is organized into two large attractor basins to which most of the trajectories converge, except for the ones starting in the intermediate density region, which converge toward a region of small and scattered attractor basins.

**Fig 3:**
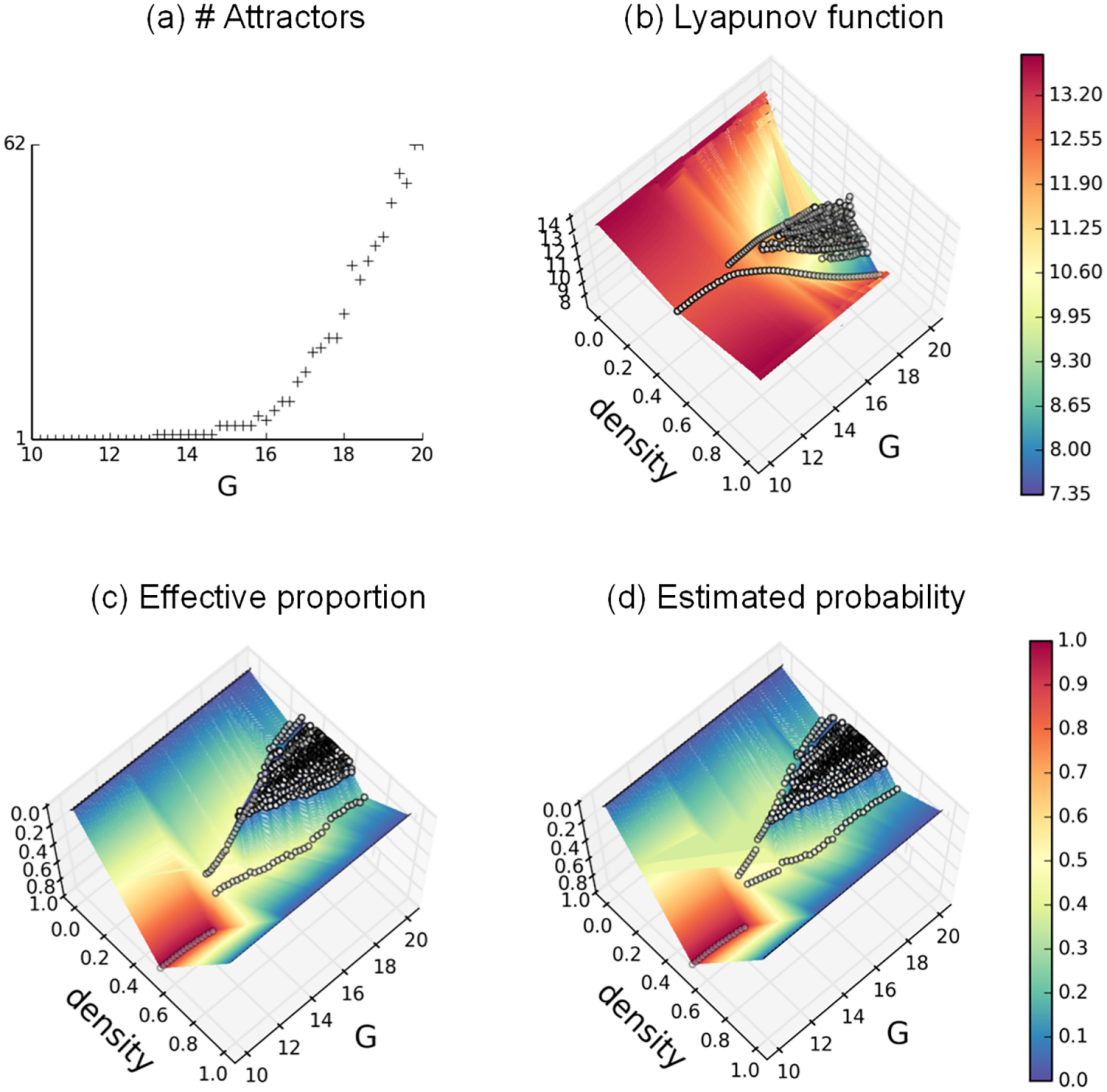
Bifurcation diagrams in the SL model. **a**. Number of attractors in function of *G*. **b**. Attractors Lyapunov values in function of attractor densities and *G*. **c**. Attractors observed proportion in function of attractor densities and *G*. **d**. Attractors predicted proportion in function of attractor densities and *G*, with *β* = 2. Each distinct attractor in subfigures **b-d** is denoted by a white dot. The color mappings, interpolated from the discrete values, are provided for enhanced readability. *P* = 1, *G* ∈ [10;20].

To better understand the transition from monostability toward multistability, we consider the cascade of bifurcations more closely. The first bifurcation, observed at *G* = 13.2, is a supercritical Pitchfork bifurcation comprising a first central attractor, which symmetrically bifurcates into two stable equilibria at the critical point. This bifurcation is rapidly followed by a second bifurcation for *G* = 14.8, giving rise to 4 different attractors. Numerous bifurcations follow for *G* > 15.6, with an exponential growth rate. We take advantage of the analytical tractability of the SL model and compute the Lyapunov function (see Eq (16)), which identifies all attractors. The evolution of the Lyapunov function is plotted for 10 ≤ *G* ≤ 20 in Fig 3b. Each distinct attractor is represented by an open circle, with its average density on the x-axis, G-value on the y-axis and its Lyapunov value on the z-axis. The two main valleys deepen for increasing *G* and correspond to the two extremal activity patterns (“up” and “down” states). The intermediary activity patterns, displaying a higher “energy” level, are less likely to be reached. The corresponding probability of appearance for each attractor is shown in Fig 3d using the Boltzmann-Gibbs distribution (see Eqs (14) and (15)), and compared in Fig 3c to their observed distribution.

#### Attractor landscape of the three network models SL, SG and DG

**Fig 4:**
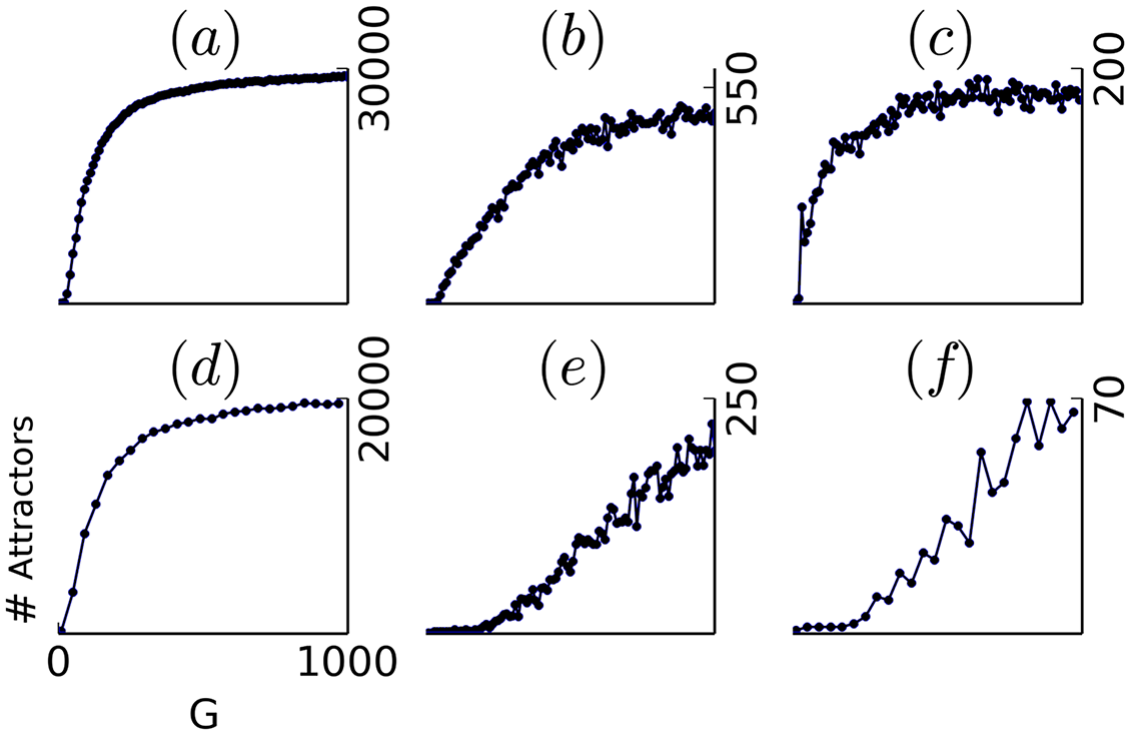
Estimated number of attractors in function of the gain G. from 33,000 random initializations and for different models and connectomes. **a,d–f**: SL model, with *P* = 1. **b**: SG model, with *P* = 0.9. **c**: DG model, with *P* = 1. panel **a-c** and **e** use the the Hagmann composite connectome [38]. panel **d** and **f** use a a DTI-based connectome extracted from an MRI image from the HCP project database [43]. panel **e** and **f** use randomized versions of the initial connectomes (**a** and **d**).

One key quantity determining the variability of the brain signal, under the influence of noise, is the number of attractors attained by the dynamics from the space of initial conditions. This number reflects the degree of multistability (or scattering of the attractor basins). The number of attractors obtained for increasing values of *G* is plotted in Fig 4 for the different models or different structural connectivity matrices. As expected from the spin-glass literature [39–42], a key role of the gain *G* in enhancing multistability is found in every model, where low values of *G* are interpreted as the “high temperature” case, and high values of *G* as the “low temperature” case. In the first row (Fig 4a–4c), the same connectivity matrix based on Hagmann [38] 998 ROI’s is used under the SL, SG, and DG models. A similar monotonic increase is observed for the three models. The SL model displays a very sharp increase of the number of attractors (approx. 500 attractors for *G* = 20, more than 1,000 for *G* = 30 etc…), with a rapid saturation (to approx. 29,000 attractors) possibly due to the limited size of our initial conditions set (33,000 different initial conditions). Despite the large number of initial conditions, every distinct initial condition seems to drive the dynamics toward a distinct final attractor, indicating a dramatic scattering of the attraction basins, and a probable underestimation of the total number of attractors. The SG model presents a more progressive increase, with a late plateau under 500 attractors for *G* > 500. An earlier plateau is observed in the DG case at much lower cardinalities, indicating a maximum of 150–175 distinct final attractors.

To obtain an insight into the dependence upon the connectome, different connectomes are used in the second row (Fig 4d–4f) for the SL model. The connectome used in Fig 4d has been extracted from the HCP project database [43], using the SCRIPTS pipeline [44]. It is composed of 1120 ROI, including sub-cortical regions. The curve is quantitatively and qualitatively similar to Fig 4a, with a final number of distinct attractors estimated at approx 20,000. In Fig 4e and 4f, the connectomes of Fig 4a (resp. Fig 4d) have been shuffled while preserving the in and out-degrees of the different nodes (Masslov algorithm—see Material and Methods). Here a much smaller number of attractors is observed, with a maximum of 250 in the Hagmann case, and a maximum of 70 in the HCP case. The gain *G* is consistently found to monotonically influence the degree of multistability both throughout models and throughout connectomes. The quantitative difference found between regular and shuffled connectomes, however, indicates a dramatic influence of the graph structure in enhancing multistability.

**Fig 5:**
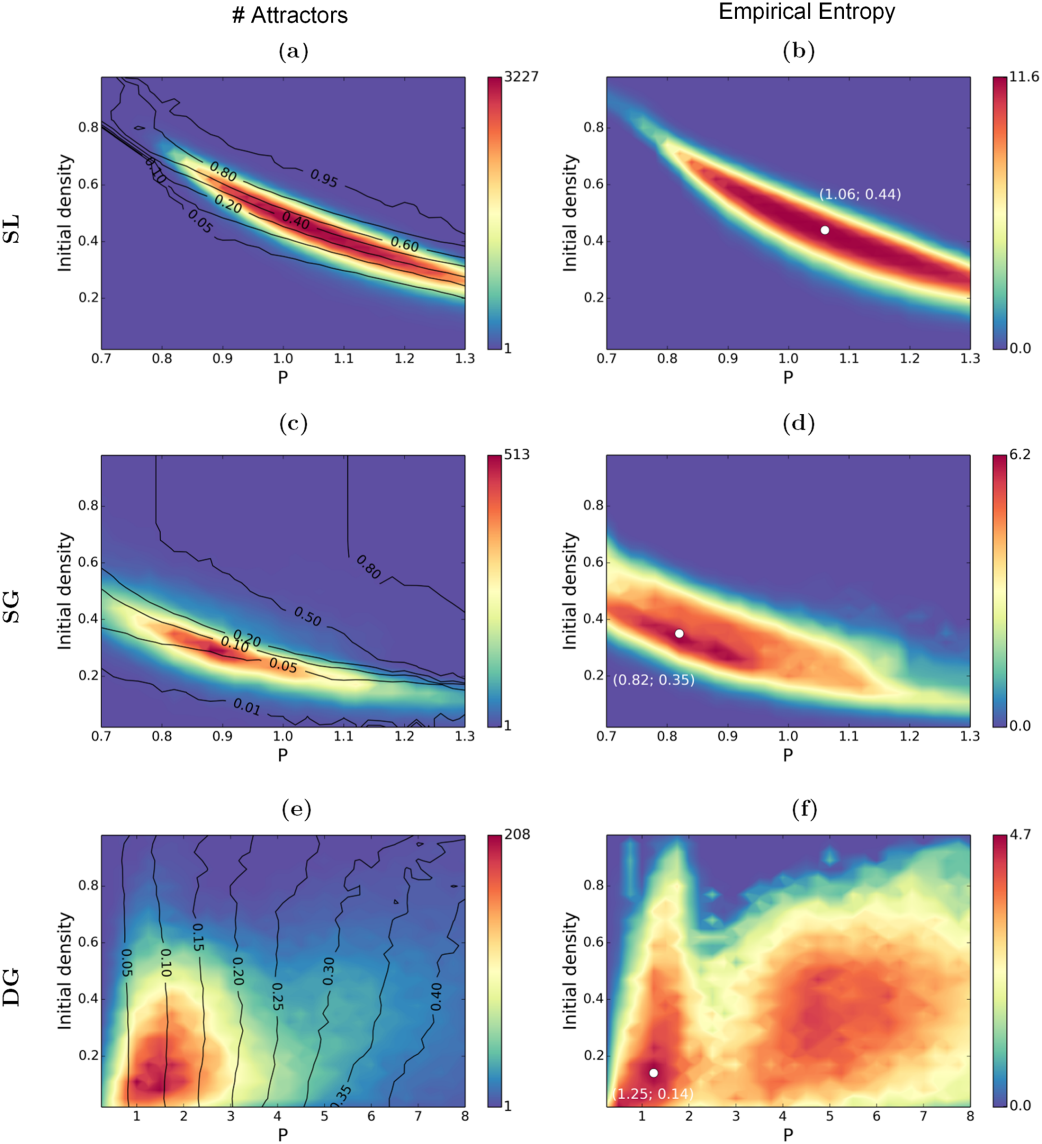
Number of attractors (left) and empirical entropy (right) in function of the scaling factor *P* and the initial density *f*_0_. 3,300 initial conditions are randomly sampled for each value of (*P*, *f*_0_). Black lines are isodensity lines corresponding to the average density of attractors. **a-b**: SL model. **c-d**: SG model. **e-f**: DG model. The white dots in **b**, **d**, **f** report the coordinates of the maximal value. *G* = 900.

Next to the gain G, the scaling factor *P* is hypothesized to play a crucial role in determining the attractor density of the models. In particular we focussed our analyses on the high gain case. Fig 5 presents the maps obtained for the three network models for *G* = 900, where we systematically varied the scaling factor *P* and the density of the initial conditions *f*_0_. For numerical reasons, only 3,300 initial conditions were sampled at each value (*P*, *f*_0_), leading to an underestimation of the number of attractors at the maxima (see Fig 4), but having no consequence on their location. Three observables are considered, namely the *number* of distinct attractors, the average *density* (average proportion of active nodes) and the *cardinality* attached to each cluster in the final set. In Fig 5a, 5c and 5e, the color code gives the number of distinct attractors at each value of (*P*, *f*_0_), and the black lines are isodensity lines obtained after calculating the average density of the final attractors. In Fig 5b, 5d and 5f, the color code gives the empirical *entropy* of the final set, based of the cardinality of the final clusters (see Material and Methods).

The two maps at the top (Fig 5a and 5b) correspond to the SL model. For low values of *P* and/or low initial densities, the system is monostable and systematically converges toward the “Down” attractor where the activity on every node remains close to zero (density < 0.05). A comparable monostable behavior is observed for high values of *P* and/or high initial densities, with the trivial “Up” attractor having a maximal activity on almost every node (pattern density > 0.9). The multistable behavior is obtained in the intermediate range (around *P* = 1, and with an initial density between 0.3 and 0.6), i.e. the system can relax to a large set of distinct attractors, with varied densities (see for instance Fig 2d–2f). The peak of multistability is attained at (*P* = 1, *f*_0_ = 0.5), where almost every final attractor is different. The empirical entropy map (Fig 5b) provides an additional insight about the predictability of the final activity patterns, where 0 corresponds to a full predictability (single output, no information). The map shows here a similar shape as the one at its left, with the highest entropy at the multistability peak. The maximal value is close to the entropy upper bound (here at 11.69, obtained with an alphabet of 3,300 symbols with equal probability). The high entropy values indicate here a varied set of equally probable final activity patterns, supporting a strong flexibility of the system.

The two maps in the middle capture the SG model (see Fig 5c and 5d). The general shape of the left map is qualitatively similar to Fig 5a, with much less attractors. The upper right corner corresponds to very dense patterns (“Up” state) and the lower left to very sparse (“Down state”). The two trivial attractors thus similarly appear to exert a strong attractivity. The multistability peak is found on a slightly different range, close to *P* = 0.9. The maximal number of attractors is close to 500, which is significantly less than in the SL case. The main difference lies in the much smaller attractor density (of the order of 0.03 − 0.1), which indicates much sparser activities than in the previous case. In this network composed of purely excitatory nodes, each sparse pattern denotes a pool of neurons sharing enough excitatory links to locally keep a self-sustained activity, but not enough to excite the rest of the network. Interestingly, the entropy map shows a wide region of maximal entropy, covering regions where a relatively lower number of attractors is observed. This reveals a complexity that is not quantitatively different when the number of attractors is close to 500 or close to 30-50.

The last maps (Fig 5e and 5f) correspond to DG model. In this model, a dynamic threshold regulates the average activity (see Material and Methods), destabilizing the two extremal patterns, and giving more “space” to the intermediate patterns. In consequence, the final density is no more dependent on the initial density, showing a clear dependence on *P*: sparse activity patterns are obtained for low values of *P*; dense activity patterns are obtained for high values of *P*. Contrary to the previous cases, multistability is robustly obtained in a large region of the parametric space, for 0.2 < *P* < 10. The multistability region is about 10 times wider than in the static thresholds cases, with a peak of multistability attained between *P* = 1 and *P* = 3. The densities obtained at the peak of multistability, ranging from 0.05 to 0.15, are much lower than in the SL case, and similar to the ones obtained in the SG case. A smaller number of final distinct patterns (less than 200) is obtained than in the SL and SG cases. When considering the entropy map, two regions of maximal variability are observed. A first maximum is found for 0.5 < *P* < 2, corresponding to low densities (around 0.1), and a second maximum is observed for 4 < *P* < 6, corresponding to higher densities (around 0.3). The maximum value is not quantitatively different at the two maxima (around 4), while the number of attractors is. This indicates, at the first maximum, that a high proportion of attractors is unlikely to be observed with only a few being effectively expressed.

The scaling factor *P*, whose general effect is to regulate the excitation, is thus found to play distinct roles across the models. In the case of a fixed threshold (SL and SG models), it helps to establish a range of “viable” activity, where the excitation can spread over limited regions, without igniting the full brain. In contrast, the DG model is very tolerant to a variable scaling factor (a variable E/I ratio), and the role of *P* is here to regulate the density of the final activity patterns, allowing to unfold attractor sets of different densities. The density of the final activity pattern is more generally an important quantity to consider. In the models having a fixed activation threshold (SL and SG), the final density is very variable, while it is tightly controlled in the DG case. In addition, all densities can coexist in the SL model, while only “sparse” activity patterns (between 0.02 and approx. 0.4) can coexist in the SG and DG models.

### Characterizing the dynamic repertoire: Attractor set analysis

Each attractor can be mapped on the cortical surface, and interpreted functionally: an attractor provides a set of nodes that are expected to “work together”, possibly reflecting an underlying brain function. These interaction effects are characterized in two ways: The first approach is via functional connectivity and matrix-to-matrix comparison, where we compute functional connectivity under variation of control parameters for the three network models and validate it against empirical data. The second approach is based upon a direct functional pattern-to pattern comparison. For the empirical data we use a set of resting-state functional MRI (rs-fMRI) time courses [45] with a total of 35 minutes recorded in two sessions per subject.

#### Functional connectivity comparison

Three types of matrices are compared here. (i) The empirical functional connectivity matrix (EFC) is computed via Pearson correlation over the full rs-FMRI dataset, resulting in a cross-subject 998 × 998 matrix considered here as the reference. (ii) The structural connectivity matrix (SC) is the structural connectome. (iii) An attractor-based functional connectivity (AFC) is calculated in the same way as EFC when multiple attractors are obtained in the simulation. This matrix is expected to reflect the co-activations (and co-deactivations) across the different attractors of the set. A direct comparison of structural connectivity (SC) and EFC yields a correlation of 0.53 (using a Gaussian resampling of the initial DSI weights). When directly correlating the binary structure of a single hemisphere (containing one when the link is present and zero when the link is absent) with the corresponding EFC values, a correlation of 0.44 is found. Those similarity values must be contrasted against the intra and inter-subject variability as observed in the rs-fMRI signal. When comparing the EFC calculated on two distinct sessions for the same subject, an average 0.58 correlation is observed, whereas for the EFC obtained for two different subjects, an average 0.32 correlation is observed. These comparisons indicate that 0.55-0.60 correlations reflect a close fit, and 0.3-0.35 correlations reflect a more scarce but still significant fit.

For each network model and each value of (*P*, *f*_0_), AFC matrices were calculated. When multiple attractors are present, the AFC matrices show a checkerboard structure that reflects the modal spatial organization of the attractors, with well-localized anatomical groups having a tendency to share activity. This checkerboard covariance organization is a correlate of the “patchy” aspect of the attractors seen on Fig 2.

**Fig 6:**
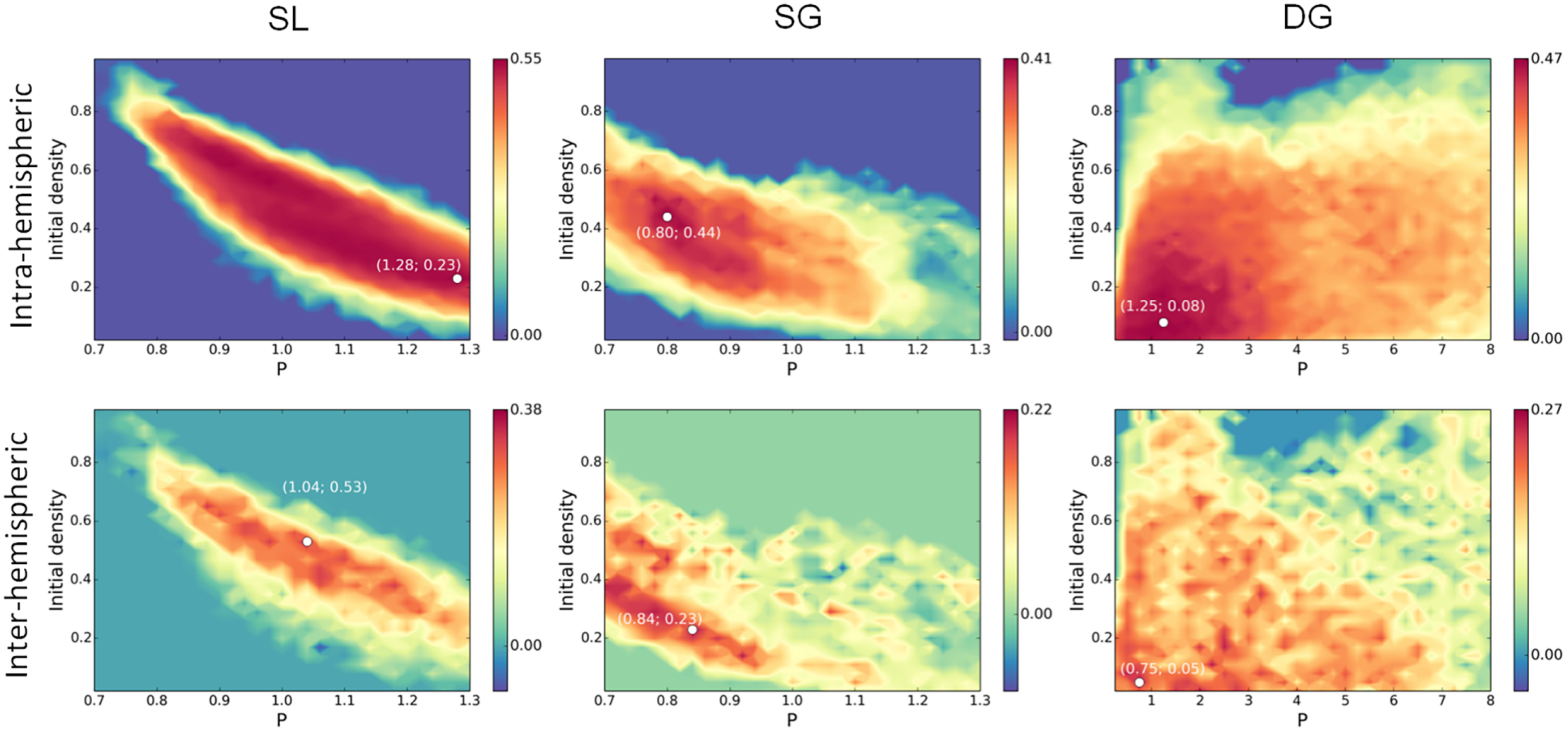
Attractor-based functional connectivity matrices are compared with the Empirical Functional Connectivity matrix. Intra- and inter-hemispheric AFC matrix values are correlated to the EFC matrix values. The comparisons are done on the different models on the same range as in Fig 5. The white dots report the coordinates of the maximal value. *G* = 900.

The correlation maps between the intra- and inter-hemispheric AFC and the intra- and inter-hemispheric EFC are shown on Fig 6. In the case of the SL model, a strong intra-hemispheric similarity (between 0.50 and 0.55) is obtained for decreasing values of *f*_0_ in the 0.8 < *P* < 1.3 range. Those maximal values are obtained along the two high and low-density lines (0.10 and 0.90 densities) and mostly reflect the local correlation structure. The best report of the inter-hemispheric correlation structure (0.32) is obtained for intermediate densities (*P* = 1.04, *f*_0_ = 0.53, around 50% nodes active). The general trend is thus a better prediction of the local correlations at low/high attractor densities, and a better prediction of the large scale correlations at intermediate attractor densities. The SL model is thus found to display a significant improvement of the functional connectivity prediction, at both local and long range, when compared with the sole structural prediction.

The similarity map obtained in the SG network model (Fig 6b) indicates a maximal correlation (0.41) at a region that is above the maximal entropy line (see Fig 5d), and corresponds to intermediate density attractors at the upper-edge of multistability (around 20-30% of the nodes active on average). The best correlation with the inter-hemispheric organization (0.22) is obtained in a region of low-density attractors, at the lower edge of multistability (around 2-5% of the nodes active on average).

When considering the DG network model (Fig 6c), the intra-hemispheric best match (0.47) is obtained for *P* < 2, on an interval that corresponds to the highest entropy (Fig 5f), with average activities ranging from 5% to 15%. The inter-hemispheric best match (0.27) is obtained on a smaller range for *P* < 1, at the leftmost edge of multistability, for a set of attractors having on average 5% of their nodes active. While slightly better than the SG model, only the inter-hemispheric correlation can here be considered as a significantly improving the prediction regarding the structural information. Thus 0.5 < *P* < 1 can be considered a relevant range for predicting the physiological activity in this model.

In summary, only the SL and DG models are found to significantly improve the EFC prediction. This increased similarity is found in the vicinity of the multistability peak, not necessary at the peak but rather close to a bifurcation line. This observation meets previous findings about a greater accuracy found at the edge rather than at the center of multistability regions [13, 14, 20].

#### Brain activation pattern comparison

Our second approach is based upon a combination of attractor sampling and clustering with the goal to gain insight into the statistics of the functional modules (or sub-networks) embedded in a connectome. Brain dynamics analysis identifies large scale networks in the rs-fMRI signal by extracting the most frequent co-activation/co-deactivation patterns. A popular approach is the use of independent components analysis (ICA) to extract the principal patterns. In contrast with the high number of attractors displayed by our model, the large-scale dynamics, as observed in the rs-fMRI signal, is known to display a relatively small set of modes, each mode reflecting known networks associated with cognition, perception and action. [2–11].

From the spin-glass literature, the number of attractors is known to combinatorially explode in the low temperature condition [39], leading to numerous “spurious” attractors. In that case, not every final attractor should represent an effective prototype, but rather a combination of them [41]. In the absence of an explicit set of prototypes, this renders the final attractor landscape quite puzzling and difficult to read. Empirical statistics must be used in order to approach the underlying prototypic organization from the attractor distribution. The idea is to identify the principal “modes” of the distribution, where a mode is a region of the sample space in which more data points are clustered showing a greater density. Dense regions of the attractor space are expected to represent larger (and thus more stable) attraction basins, and reflect the putative prototypic organization of the connectome structure.

**Fig 7:**
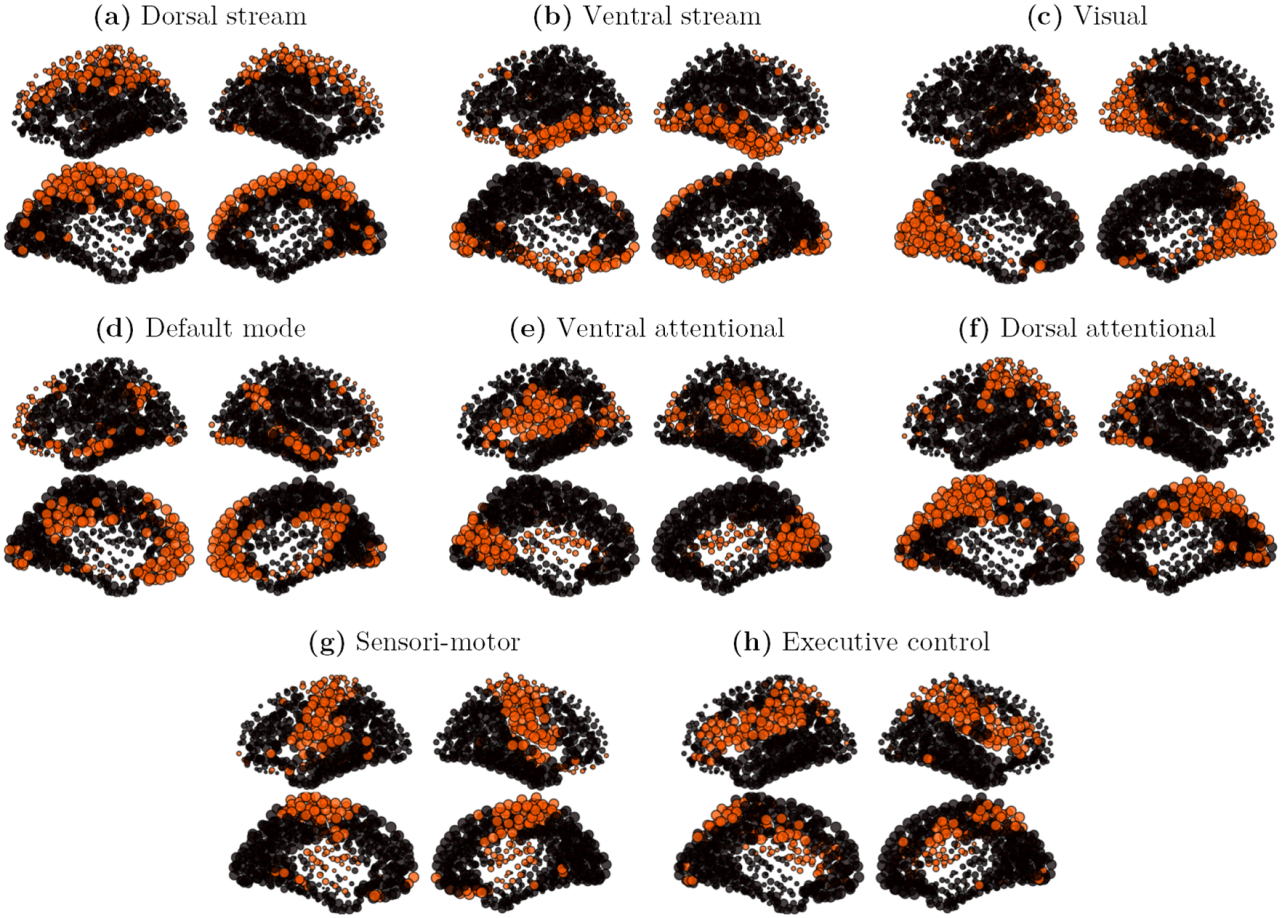
Cores of the 8 principal clusters, on multi-subject rs-fMRI signals. The initial set contains 5,200 binary observation vectors. The rs-fMRI signal is thresholded (*s* > 2). The dataset is made of 5 subjects ×35 minutes. The clusters are obtained by a double-pass clustering, with inclusion match similarity (*k* = 0.5). The cores are defined as the 25% most active nodes in a cluster. **a**: core of cluster 1 (928 observation vectors); **b**: core of cluster 2 (617 observation vectors); **c**: core of cluster 3 (528 observation vectors); **d**: core of cluster 4 (382 observation vectors); **e**: core of cluster 5 (236 observation vectors); **f**: core of cluster 6 (191 observation vectors); **g**: core of cluster 7 (163 observation vectors); **h**: core of cluster 8 (143 observation vectors).

In order to allow comparison with our attractor sets, a specific clustering algorithm was applied. The general idea is to consider each observation vector from the rs-fMRI signal as representing a particular attractor of the underlying brain dynamics. 5,200 observations vectors were extracted from the five subjects time courses of the Hagmann rs-fMRI database. A double-pass clustering, using inclusion match similarity (see Material and Methods), was applied on the observation vectors set, with a similarity threshold *k* = 0.5. 601 clusters were extracted, with cluster size strongly varying in size, the biggest cluster gathering 928 elements (17.8%), and many small ones containing only 1 element. Not every cluster being considered significant, only the characteristic vectors of the 8 principal clusters, ranked by importance, and gathering 61% of the signal, are presented on Fig 7, where the characteristic vectors are here defined as the 25% most active nodes of the cluster. All patterns of the set spread on a large scale, displaying strongly bilateral and symmetrical components. Our set of patterns contains (a) a superior and medial prefrontal mode, including superior parietal elements (Dorsal stream, 928 observations), (b) an orbito-frontal mode including inferior temporal and para-hippocampal components (Ventral stream, 617 observations), (c) a medial and lateral occipital mode corresponding to the visual network (528 observations), (d) a centro-medial mode with inferior parietal and temporal components corresponding to the default mode network (DMN, 382 observations), (e) a superior temporal and ventral prefrontal mode including a primary visual component (Ventral attentional, 236 observations), (f) a lateral occipital mode including superior and medial parietal components (Dorsal attentional, 191 observations) (g) a pre and post-central mode corresponding to the sensori-motor network (163 observations), and (h) a superior parietal and dorso-lateral prefrontal mode corresponding to the fronto-parietal executive control network (143 observations). Those components are clearly delineated and consistent with the literature [2–7, 9].

To address the question how much of the large functional networks are predicted by the activation patterns obtained in our simulation-based attractor sets, we validate the simulated attractor sets against empirical data. We focus in particular on a detailed comparison of the SL and DG network model.

#### SL model

**Fig 8:**
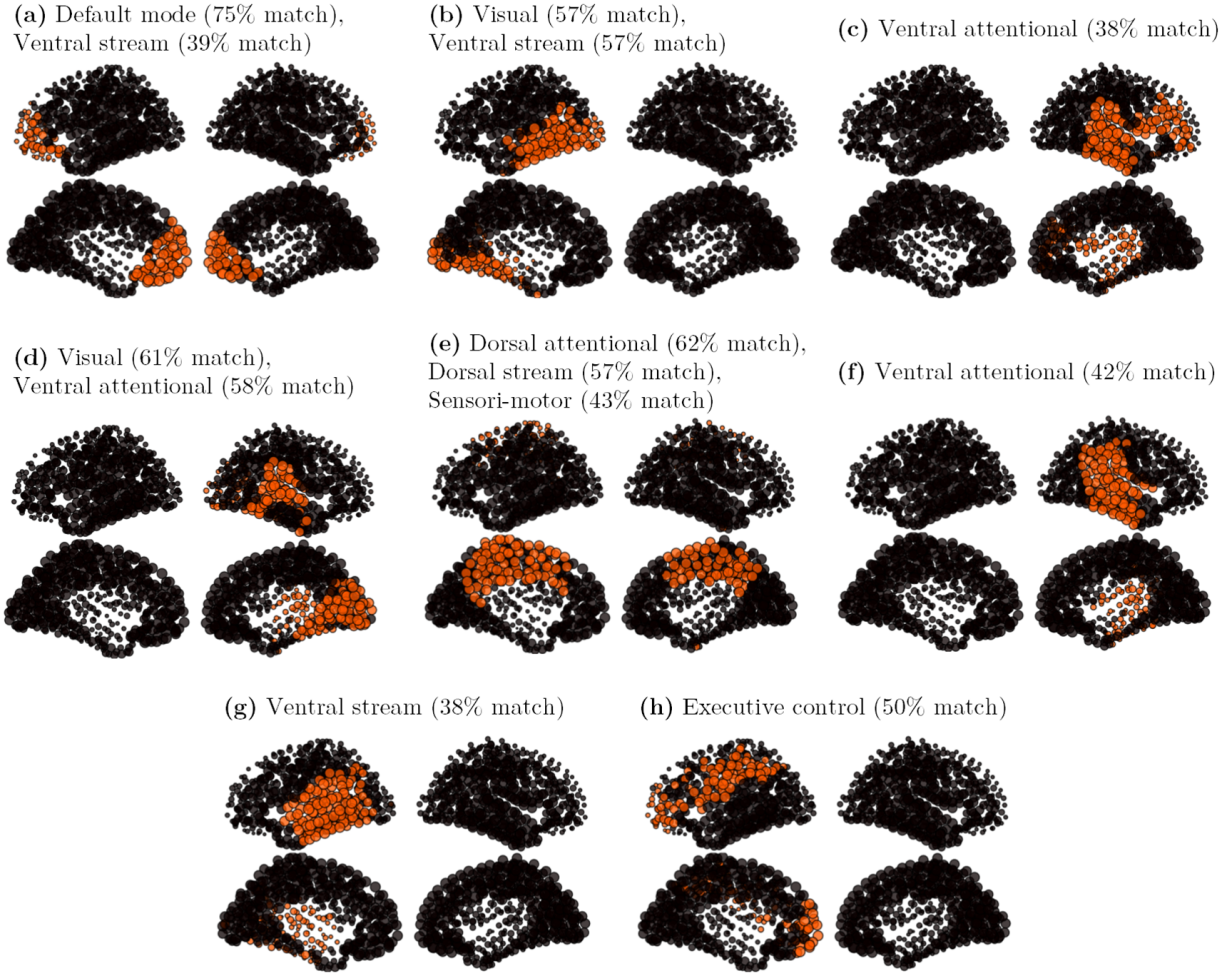
Cores of the 8 principal clusters, on a SL attractor set. The initial attractors set contains 28,849 attractors, with *P* = 1 and *G* = 900. The clusters are obtained by a double-pass clustering, with inclusion match similarity (*k* = 0.8). The cores are defined as the 10% most active nodes in a cluster. **a**: core of cluster 1 (4,089 attractors); **b**: core of cluster 2 (3,048 attractors); **c**: core of cluster 3 (2,045 attractors); **d**: core of cluster 4 (1,573 attractors); **e**: core of cluster 5 (907 attractors); **f**: core of cluster 6 (892 attractors); **g**: core of cluster 7 (881 attractors); **h**: core of cluster 8 (844 attractors).

A first set of patterns is built from the graded-response Hopfield model (SL model) using 33,000 random initial conditions. The attractor set, composed of 28,849 attractors, displays dense activity patterns with on average 50% of the nodes active. Each attractor seems to be composed of several anatomical “patches”, and the same patches seem arbitrarily active (or inactive) across the different attractors. Significant co-activations do not show up at first sight. A double-pass clustering, using inclusion match similarity (see Material and Methods), was applied over the first set of 28,849 attractors, with a similarity threshold *k* = 0.8. A total of 725 clusters was found, with cluster size strongly varying in size, with the biggest cluster gathering 4,089 similar elements (14.2% of the total), and many small ones containing only 1 element. Half of the nodes being on average active in the SL model, repeatedly active nodes in a cluster identify the cluster core: characteristic vectors are here defined as the 10% most active nodes in a cluster. The characteristic vectors of the 8 principal clusters, ranked by importance, and gathering 50% of the total attractors of the set, are presented on Fig 8. From this set, 2 patterns (a and e) present a bilateral symmetry, 3 patterns (b, g and h) are left-lateralized and 3 patterns (c, d and f) are right-lateralized.

Despite the strong core selection, a widespread organization of the patterns is observed. The different patterns are constituted of several well-delineated, but not necessarily contiguous, anatomical regions. Each cluster core should thus be seen as predicting a typical large-scale interaction pathway, either within or across the hemispheres. The logic of the comparison is thus identifying which *parts* of the functional organization are correctly reported by the simulation, and which parts of it differ. For this purpose, an “inclusion match” similarity is used which indicates which proportion of a cluster core (10% most active nodes) correctly match with the 25% most active nodes of a rs-fMRI-based cluster. This match is considered significant when the proportion of correct predictions is greater than 37% (t-value > 3), good when the proportion is greater than 51% (t-value > 6), and strong when the proportion is greater than 65% (t-value > 9). All patterns show a significant correspondence with at least one functional pattern of Fig 7.

The first cluster core (Fig 8a, 4,089 attractors) links bilaterally the frontal pole, the orbito-frontal cortex and the anterior prefrontal cortex, displaying a strong match with the anterior part of the Default-mode network. The second cluster core (Fig 8b, 3,048 attractors) is a left-lateralized pattern linking the medial and lateral occipital cortex with the posterior temporal cortex (left ventral visual pathway) displaying a good match with the visual and the ventral functional networks. The third cluster core (Fig 8c, 2,045 attractors) is a right-lateralized pattern linking superior temporal and inferior parietal regions with the ventral prefrontal cortex. A fair match with the ventral attentional network is observed. The fourth cluster core (Fig 8d, 1,573 attractors) is the right contralateral homologous of Fig 8b (right ventral visual pathway), displaying a good match with the visual and the ventral functional networks. The fifth cluster core (Fig 8e, 907 attractors) is a centro-medial pattern, linking bilaterally the mesio-parietal and cingulate cortex. The sixth cluster core (Fig 8f, 892 attractors) is a right-lateralized pattern, linking the superior temporal and the inferior parietal regions, showing a fair match with the ventral attentional network (similar to Fig 8c except for the ventral prefrontal regions). The seventh cluster core (Fig 8g, 881 attractors) is the left contralateral homologous of pattern Fig 8f, showing a fair match with the ventral stream. At last, the eighth cluster core (Fig 8h, 844 attractors) is a left-lateralized pattern linking the lateral parietal regions with the ventral prefrontal cortex and displaying a consistent match with the executive control network. Two main differences with the rs-fMRI functional networks can be highlighted here. A first difference is the ventral visual pathway here separated in a left and right components (b and d), while separated in bilateral visual and ventral components in the rs-fMRI signal analysis (b and c). Both interpretations can however be considered as physiologically relevant. On contrary, the patterns (c), (f) and (g) seem relatively specific to simulation, for they report a “vertical” network, from the inferior parietal to a large portion of the temporal cortex (except the temporal pole), that has no obvious counterpart in physiology. The non-contiguous link between the inferior parietal regions and the inferior prefrontal regions, reported in pattern (c) is however consistent with the non-contiguity of the executive control network.

#### DG model

A second set of attractors is built from simulations of the DG network model with *P* varying from 0.5 to 7 (with 0.25 steps), providing a set of 36,459 different attractors. This second set of attractors is more regular, the attractors are much sparser than the one generated by the SL model. The same clustering algorithm was applied over this second set of attractors. A total of 80 clusters was found, with an even stronger cluster size imbalance, with the biggest cluster grouping 11,141 similar elements (30.6% of the total), and the many tiny ones having only one element like previously. A small number of configurations thus tends to dominate the dynamics here.

**Fig 9:**
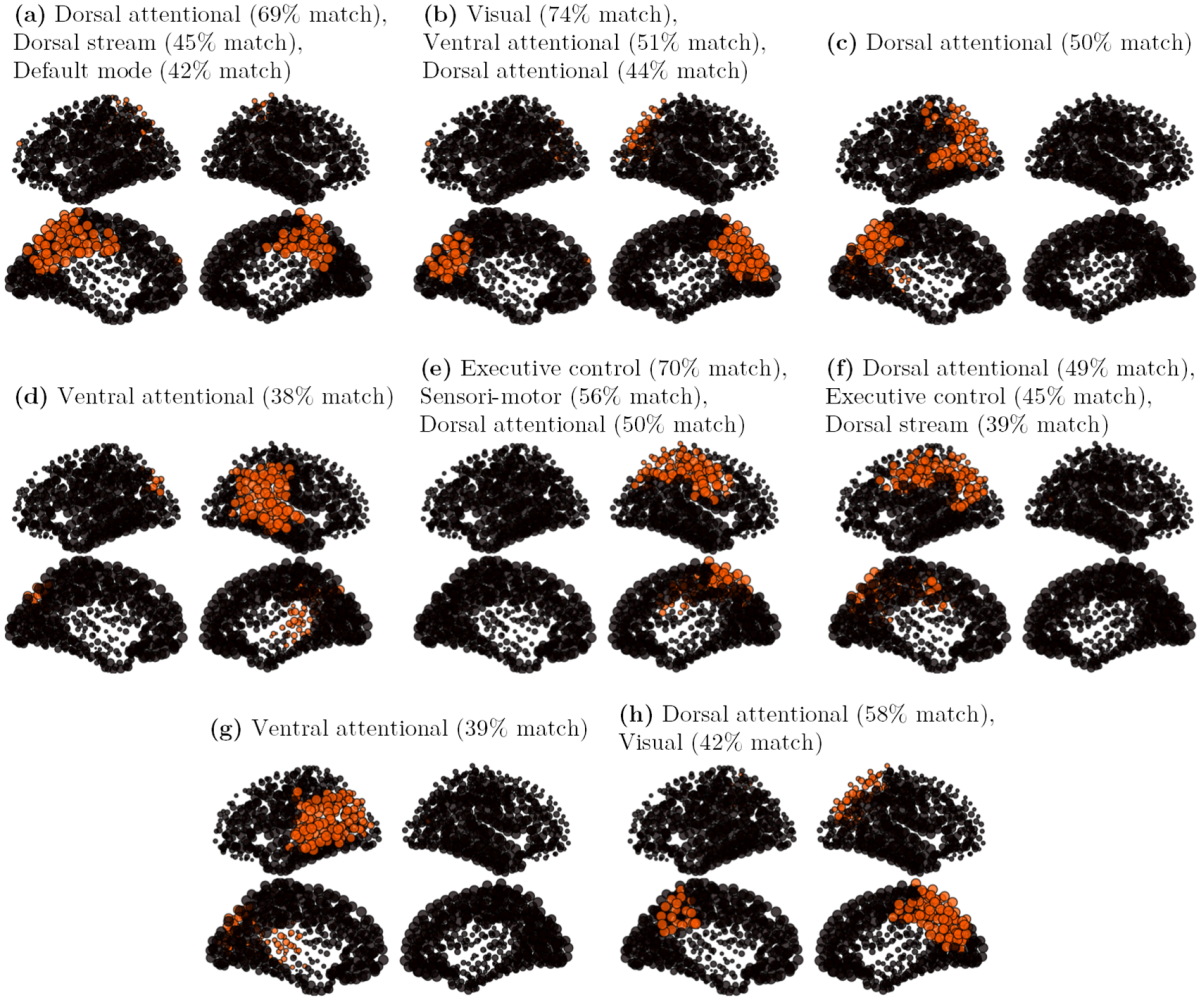
Cores of the 8 principal clusters, on a DG attractor set. The initial attractors set contains 36,459 attractors, with *P* ∈ [0.5;7] and *G* = 900. The clusters are obtained by a double-pass clustering, with inclusion match similarity (*k* = 0.8). The cores are defined as the 10% most active nodes in a cluster. **a**: core of cluster 1 (11,141 attractors); **b**: core of cluster 2 (6,376 attractors); **c**: core of cluster 3 (5,494 attractors); **d**: core of cluster 4 (2,833 attractors); **e**: core of cluster 5 (2,832 attractors); **f**: core of cluster 6 (1,347 attractors); **g**: core of cluster 7 (1,275 attractors); **h**: core of cluster 8 (1,236 attractors).

The characteristic vectors of the 8 principal clusters, ranked by importance, and gathering 89% of the total attractors of the set, are presented on Fig 9. The characteristic vectors are here again defined as the 10% most active nodes in a cluster, and an “inclusion match” comparison with the eight most characteristic rs-fMRI-based patterns (Fig 7) is done. From this set, 3 patterns (a, b and h) present a full or partial bilateral symmetry, 3 patterns (c, f and g) are left-lateralized and 2 patterns (d and e) are right-lateralized. The first cluster core (Fig 9a, 11,141 attractors) links bilaterally the cuneus, the paracentral lobule and the posterior cingulate. This centro-medial pattern is sometimes considered as the functional core of the Default mode network [46], while also matching with the dorsal attentional network in our analysis. The second cluster core (Fig 9b, 6,376 attractors) links bilaterally the cuneus, lingual and the inferior part of the precuneus, displaying a strong match with the visual network. The third cluster core (Fig 9c, 5,494 attractors) links the left inferior parietal region with the cuneus through the superior parietal cortex, displaying a fairly good match with the Dorsal attentional network. The fourth cluster core (Fig 9d, 2,833 attractors) links the left inferior parietal region with the superior temporal region, showing an incidental match with the ventral attentional network. The fifth cluster core (Fig 9e, 2,832 attractors) links the left superior parietal region with the post-central sulcus, showing a good match with the executive control and dorsal attentional networks. The sixth cluster core (Fig 9f, 1,347 attractors) is the contralateral homologous to Fig 9e. The seventh cluster core (Fig 9g, 1,275 attractors) is the contralateral homologous to Fig 9d. At last, the eighth cluster core (Fig 9h, 1,236 attractors) is a leftmost homologous of Fig 9a, showing here again a good match with the dorsal attentional network. In that model, the frontal and orbito-frontal regions are only present in smaller clusters not represented on the figure. The most prominent regions are here the precuneus, the inferior parietal region, the bank of the superior temporal sulcus, and the post-central sulcus.

In summary, the SL model, with a more widespread distribution of activity, provides a greater variety of attractor patterns. In particular, the ventral visual stream and the orbitofrontal and medial prefrontal network, that regularly show up in the SL model, are barely present in the DG model. On the contrary, centro-medial as well as superior and inferior parietal activations, characteristic of the dorsal stream and dorsal attentional networks, seem to dominate the DG attractor sets. Like in the SL model, a non-physiological “vertical” parieto-temporal network, having maybe closest resemblance with the ventral attentional network, is found. An explanation for this functional network maybe the overestimation of the parieto-temporal links in the initial tractography, or an activation imbalance due to underestimation of links in the connectome (like the callosal fibers for instance, see also [47]). Bilateral large-scale and non-contiguous functional networks, like the Default mode network [48], are not reported in full by the simulation. These results thus only provide clues about large-scale affinities. Some of them, like the visual ventral stream (Fig 8b and 8d), or the non-contiguous executive control network (Fig 8h), are not obvious in the analyses.

### Exploring the dynamic repertoire: Functional Connectivity Dynamics (FCD)

In this section we present evidence for the non-stationary spatiotemporal dynamics in the three network models and highlight their differences. We first present typical noise driven time courses obtained in regions of high multistability of the three network models considered. Then we present a windowing approach to the estimation of the non-stationarity of their spatiotemporal dynamics, called the functional connectivity dynamics (FCD). We use this metric to show correspondence between the real fMRI time courses and the simulated ones.

**Fig 10:**
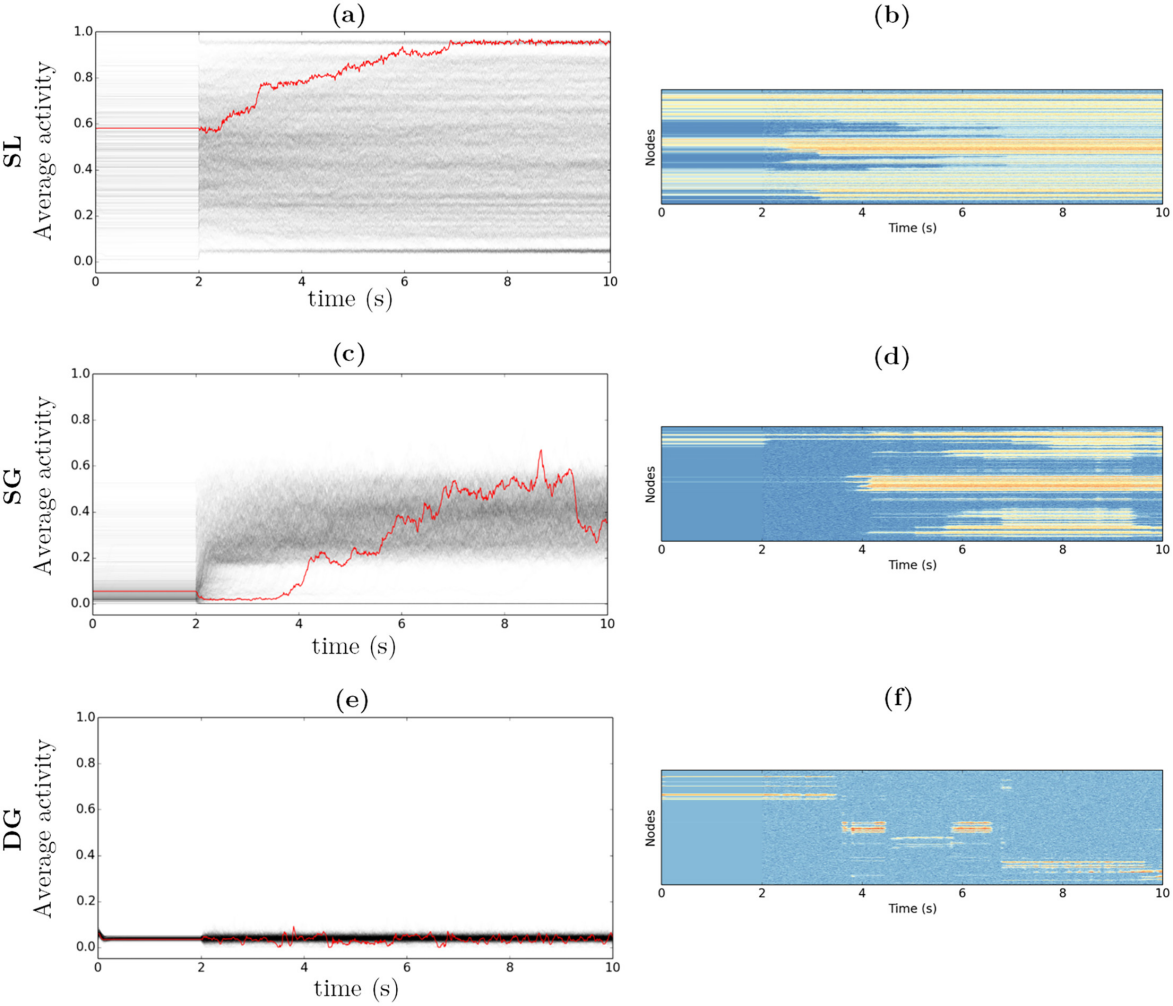
Noisy dynamics time course. The dynamics is noiseless for 2 s, and noisy for the rest of the simulations. **a**, **c**, **e**: gray lines: 1,000 average time courses—red line: sample average time course. **b**, **d**, **f**: sample spatio-temporal time course. Color code: from blue (low potential) to red (high potential). *G* = 900, *τ*_*x*_ = 10 ms, *τ*_*θ*_ = 80 ms, *σ*_*x*_ = *σ*_*θ*_ = 0.2. **a**, **b**: SL model, *P* = 1. **c**, **d**: SG model, *P* = 0.86. **e**, **f**: DG model, *P* = 0.6.

We study in Fig 10 the temporal behavior of the noise-driven extension of the 3 network models (Eqs (6)–(9)), in the parametric range where a prominent multistability is expected (namely the regions of the parameter space dispaying the highest entropy—see Fig 5). We separate the time scales of the activity and the thresholds, with *τ*_*x*_ = 10 ms and *τ*_*θ*_ = 80 ms. The thresholds dynamics is thus slower than the activation dynamics, making possible the emergence of more complex dynamics. We choose a moderate level of noise (*σ*_*x*_ = *σ*_*θ*_ = 0.2) in order to have the attractor basins “close enough” to the ones obtained in the deterministic case. Each simulation lasts 10 seconds: in the first 2 seconds, the dynamics is deterministic; then the noise is turned on for the remaining time (from 2 s to 10 s). For each dynamical system, 1000 initial conditions are chosen among the set of final attractors obtained after random sampling search (see previous sections). Each gray line corresponds to the time course of the average activity for a different initial condition. The red line corresponds to a particular time course we picked up for its large temporal variability. The corresponding 998-nodes spatiotemporal time courses of the node activations are shown below in color code.

The SL model (see Fig 10a and 10b) generates overlapping noisy trajectories forming a fuzzy cloud that covers almost the totality of the [0, 1] interval, reflecting the strong variability of the average activities. The darker the gray, the stronger the density of trajectories. The two dark lines at the high and low levels of activity indicate a significant attraction toward the “Up” and “Down” states. Intermediate trajectories are however maintained, with random excursions around the varied intermediate patterns. The red time course represents a typical multistable trajectory. Several steps are observed that systematically push the trajectory toward stronger levels of activity, finally reaching the “Up” state and remaining on it. The spatio-temporal time course (Fig 10b) displays the progressive recruitment of more and more nodes, issuing a pattern with most of its nodes active. Other observations of temporally multistable trajectories confirm the systematic drift toward one of the two trivial attractors. Either by recruiting new nodes, the dynamics evolves toward the high activity state, or by progressive extinction, the dynamics evolves toward the low-activity regime with nodes displaying a noisy activity at low rates. Long-lasting temporal multistability appears thus difficult to implement in this model. Despite their number and variety, the intermediary attractors appear not stable enough to maintain a realistic switching activity in the long run.

The simulations of the SG model (see Fig 10c and 10d) show a slightly different picture. Most of the initial conditions correspond to very sparse (i.e. very local) activation patterns. The introduction of noise at 2 seconds leads to a strong remapping of the activity, with strong contrast against the set of attractors obtained by sampling. In some cases, the dynamics converges toward the “Down” state and remains stuck in it (lower line). In most of the cases, the average activity gradually increases toward an average activity between 0.2 and 0.5. No tendency toward higher activities is observed, but rather different spatial patterns assembling and disassembling over time (see Fig 10d), A strong core activity in the centro-medial cortex is observed (cuneus, precuneus, paracentral lobule, posterior cingulate…), with a possible occipital and inferior parietal component, and a variable number of nodes activated in the rest of the cortex (with an apparent tendency toward bilateral activation).

The DG model shows a consistent multistable regime for various values of *P* (see Fig 10e and 10f). The most temporally variable trajectories are obtained for *P* = 0.6, which corresponds to a very sparse level of activation (around 0.05) and to the leftmost limit of the multistability region (see Fig 5). Every initial condition results in an apparent low activity regime. When considering a particular trajectory (red trajectory), small excursions to the “Down” activity are observed. When spatially displayed (Fig 10f), distinct spatial patterns, with similar density, are visited during a single trajectory. Because of the low amplification, the activity is prone to fall to the “Down” state, which plays the role of a reset facilitating excursion toward a new attractor basin.

**Fig 11:**
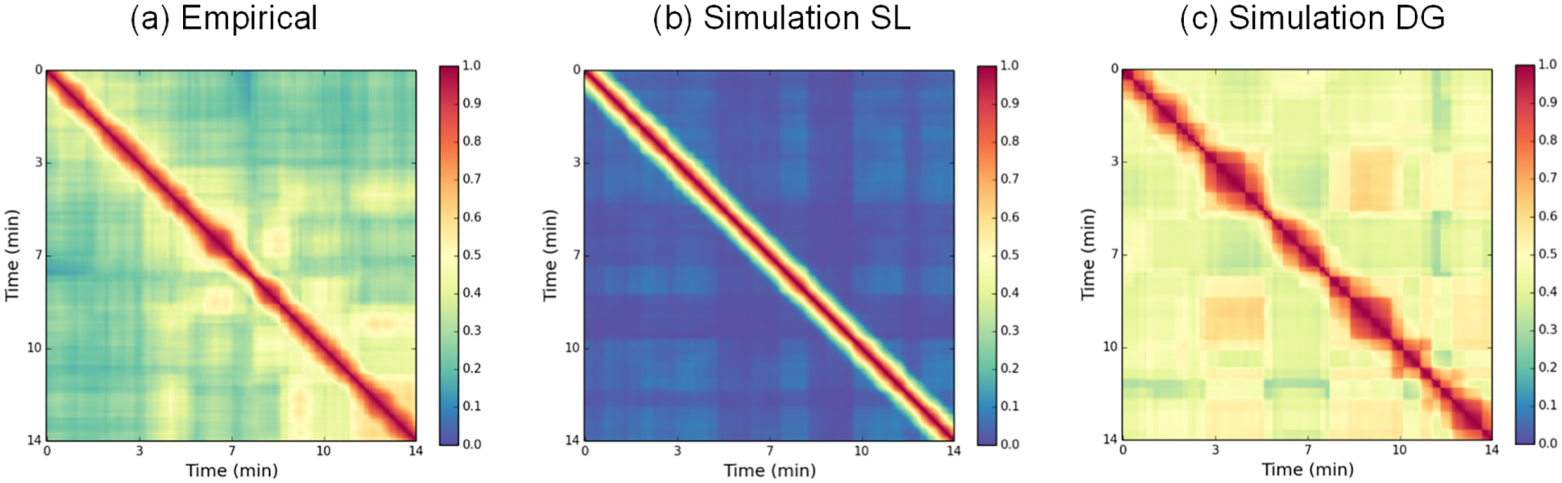
Functional Connectivity Dynamics matrices. **a**: from the empirical BOLD rs-fMRI data of one healthy subject (15 min session). **b**: from a 15 min simulated BOLD signal on the SL model, with *P* = 1, *G* = 12.6 and *σ*_*x*_ = 0.1. **c**: from a 15 min simulated BOLD signal on the DG model, with *P* = 0.75, *G* = 900, *σ*_*x*_ = *σ*_*θ*_ = 0.04. Time window *ω* = 1*min*.

As proposed in [13], the FCD is measured by the change in Pearson correlation between time-shifted FC matrices in a session (see Material and Methods). It is a synthetic measure of the underlying non-stationarity, which captures the global FC dynamics on the network level, but by construction overlooks non-stationary behavior between individual node pairs, when averaged across all node pairs. FCD thus quantifies the stationarity and persistence of the set of functional links as a whole and provides insight in the degree of the global functional organization of the network. A FCD matrix is computed for a single subject’s rs-fMRI signal and is compared in Fig 11 to two synthetic FCD matrices generated from the SL and the DG models. In the physiological FCD (Fig 11a), each row shows a typical fluctuation of correlation across the time axis with regards to the temporal reference on the diagonal, with alternations of high (≃ 0.5) and low (≃ 0.3) correlations on large temporal intervals. Those alternations were also obtained in simulation in very specific parametric ranges on the SL and DG models. In the SL model, it is observed at the critical gain *G*_*c*_ = 12.6. When computing the fMRI signals (see Methods), long-lasting alternations between the “up” and “down” patterns are observed in the resulting FCD. However, the low correlations observed outside the diagonal (≃ 0.15), reflect unrealistic changes in the FC organization across time. Similar FC variations are observed in DG model for a large span of *P* values (*P* ∈ [0.5;2.5]). The most robust alternating behavior is obtained around the lowest bifurcation (*P* = 0.75, see Fig 11c). For moderate levels of noise, a more constant FC organization is maintained across time, with correlation values alternating between ≃ 0.3 and ≃ 0.6. The analysis of the FCD thus shows that realistic ultra-slow alternations of invariant epochs of stationary FC (resulting in checker board patterns) can be observed in both models. The parameter ranges, for which these checker board patterns in the FCD appear, are however limited, near to a critical value in both cases (i.e. *G* = 12.6 on the SL model and *P* = 0.75 in the SL model). The low recurrence of FC states observed in the SL dynamics moreover indicates a closer correspondence of the empirical data with the DG model. The general trend is thus a better realism of the DG model when comparing the simulated time courses with the real ones.

## Discussion

In the present study, we have systematically analyzed the capacity of connectome-based networks to store network patterns and create sets of multistable fixed point attractors. To generalize our analysis beyond traditional synaptic excitatory and inhibitory couplings, we have integrated coupling-mediated effects of altered excitability (dynamic threshold adaptation) into the network modeling. Although these effects are here treated exclusively phenomenologically (i.e. not based upon a biophysical mechanistic derivation), their consequences are well-known to be relevant for spatiotemporal network propagation and thus cannot be ignored. For instance, spatiotemporal phenomena such as slowly propagating depolarization waves and epileptic seizure propagation are closely linked to extracellular activations involving glia [35] and feeding back on the overall excitability of a neuronal population. Proix et al [44] took a similar approach in the discussion of the dynamics of two epileptogenic neural populations. To at least phenomenologically integrate such effects upon the network organization, we considered three variants of the graded-response Hopfield model, in which non-traditional coupling effects are absorbed as coupling-mediated influences upon two of the Hopfield parameters, gain G and scale P. When coupling such generalized Hopfield models through a connectome, we found indeed major effects upon the number of attractors, their empirical validation against fMRI data, as well as their degree of non-stationarity. We discuss the three key findings in the following.

### The connectome structure enhances multistability

Our attractor sampling approach identifies the parametric range, under which a robust multistable behavior is obtained. Large attractors sets are obtained at the high gain (low spin-glass temperature) condition, every node having its activity either close to 0 or 1. This large number of static attractors exceeds by several orders the size of attractor sets generally reported in connectome based simulations [20, 42]. The number of attractors in a given network model may subserve the network capability to attain various functional configurations [49], which has been termed the dynamic repertoire [14, 50]. Previous works on the resting state dynamics that have explicitly extracted and counted network states have been typically concerned with equilibrium states of the network [13, 20]. Here we have explored the network mechanisms influencing the network’s capacity to store patterns. Furthermore, we demonstrated that the number of attractors can be expanded by four orders of magnitude beyond the so far reported number of patterns, and that this attractor state can indeed be explored when driven by noise. The distinguishing feature of our model to other models is the local threshold setting, introducing a symmetry that helps the system to visit every possible stable configuration, as well as the formal separation of the model’s free parameters into a gain (*G*) controlling the node excitability and a scaling factor (*P*) controlling the excitatory/inhibitory ratio. Those two parameters were found to independently control the multistability, with a cascade of bifurcations of the Pitchfork type observed when controlling *G*, and a non-monotonic behavior, reminiscent of the more detailed mean-field models behavior [20], when controlling *P*. We moreover show this strong multistability to be consistently obtained on different connectome datasets, while considerably weakened when using randomized versions of the initial connectomes. This finding supports the idea of an intrinsic small-world/scale-free structure of the connectome [38, 51, 52], providing a support to the multistability, as already suggested by [42].

### Model-independent fixed-point dynamics segregates the connectome into distinct resting-state networks

Clear spatially-segregated components are identified from the distribution of the attractors. Sets of principal modes are obtained, in different proportions, across the different models, using a specific “inclusion match” clustering analysis. The strongest complexity in number, variability and spatial extent is obtained on the original Hopfield graded-response (SL) model. Our clustering analysis reports specific large-scale networks, displaying a strong similarity with the independent components [2, 4] or communities [8] obtained from fMRI resting state time course analysis. Some of them (frontal pole core, centro-medial core, primary visual core) were already identified in graph-theoretical approaches [38], while others, like left and right ventral visual streams, or a left-lateralized parieto-frontal network, were not observed in the graph-theoretical approaches. In contrast, adaptive-threshold based variants of the Hopfield model (SG and DG), although displaying sparser activity patterns, provide a lesser variability, with the centro-medial component dominating the samples, representing between 50 and 75% of the total attractor sets. This central activity is reminiscent of the default mode network [48], although the orbitofrontal component remains absent. The other modes include a primary visual modality, a superior parietal element, a pre/post-central medial element, and a superior-temporal element. Although stemming from different principles, those different modes are similar to the “regional modules” identified in [38]. Attractor cross-correlation matrices were compared with the rs-fMRI-based functional connectivity matrices, also providing contrasted results when comparing the different models, with the SL model presenting the advantage of predicting both the intra-and inter-hemispheric functional connectivities. A first conclusion from these comparisons is the more flexible and more widespread-distributed attractor sets in the SL model, providing a better account of the large-scale structural organization than models having a tighter control of the nodes average activity.

### Global dynamic feedback improves the realism of the reconstructed time courses

When noise is introduced into the network, the functional network dynamics becomes non-stationary, demonstrating epochs of invariant functional connectivity and transitions between them. Noise-driven exploration of the brain’s dynamic repertoire has been hypothesized to play an important role in the execution of cognitive functions and serve as a biomarker of aging [15] rendering the relation between network capacity and noise strength significant. Noise causes a large proportion of attractors to vanish and become invisible, leaving space to a much smaller attractor sets, including trivial attractors like the “Up” (full brain activation) and “Down” (full brain deactivation) sets. In the absence of density control, a “centrifugal” tendency toward either the “Up” or “Down” state is observed. Only the density control case (DG model), imposing stable density across time, provides a condition where no tendency toward over-activation or extinction is observed. Only this central control of the average activity seems capable of maintaining the checker board pattern of FCD (transitions between longer lasting epochs of invariant FC) in a parametric range close to the bifurcation point. The existence of a central control in the brain is of course highly conjectural. In some studies the role of the thalamus or the claustrum is hypothesized to coordinate distant synchronized activities [53, 54]. Nevertheless, our findings demonstrate that a dynamic feedback mechanism (here through the dynamic adaptation of the thresholds) has the capacity to significantly enhance the complexity of the time courses and qualitatively capture the non-stationary behavior of brain networks at rest.

In conclusion, we propose a novel approach to large-scale brain simulation that encompasses the capability of generating large sets of spatially-distributed attractors, reminiscent of well-known resting state networks, and theoretically interpreting the parameters that control the dynamics. Apparent differences in the final distribution of activities are found in case of local or global control of the nodes activity. More precisely, physiologically plausible switching dynamics of the functional connectivity is obtained under a global control of the average activity (DG model). The multistable behavior is obtained over a large parameter range, but the best fit with the ultra-slow functional connectivity dynamics, as observed in the rs-fMRI time courses, is found at the edge of multistability, a parameter region that also corresponds to the highest entropy of the attractors. Our work highlights the importance of the noise-free dynamics in analyzing the attractors’ landscape, for identifying high-multistability/high entropy parameter regions that both fit with the most physiological distributions of activity, and the most relevant time courses in the noisy condition.

## Materials and Methods

### Structural connectivity

To develop large-scale brain models we use a structural connectivity matrix, the Connectome, obtained from tractographic reconstruction of Diffusion Spectrum Imaging (DSI) data of five healthy subjects [38]. Diffusion MRI provides information on fiber orientations in vivo with anisotropic diffusion of water in the brain. The anisotropy is mainly caused by the barrier created by myelin sheath insulating neurons axon. 998 regions of interest (ROI) are extracted from a parcellation applied on a standard MRI. The resulting connectome is averaged from the most significant 10,000 connections of all five individual connectomes after a k-Core decomposition (details are in [38]). The final Connectome is symmetric with positive weights. Null models are generated using a randomization algorithm (Maslov and Sneppen [55]) that preserves the degree of the node (random switch of the non-null edges).

### Functional connectivity dynamics (FCD)

We compute the functional connectivity for windowed portions of the rs-fMRI time series and then calculate the Pearson correlation for time-shifted FC matrices. The FCD is visualized as a correlation matrix over the time shifted values. Three models are considered: “Static and Local” (SL) threshold model (equivalent to the traditional Hopfield network node), the “Static and Global” (SG) threshold model, and the “Dynamic and Global” (DG) threshold model. Time series of 15 minutes were computed on the SL and DG models at 998 nodes resolution. The signals were convolved with a Balloon/Windkessel kernel [56–58], resulting in a BOLD time course with 0.5Hz resolution. Then a 1 minute sliding window is used to generate a time series of Pearson correlation values for each link, resulting in a *N* × *N* × *T* matrix, where *T* equals 14min (total duration minus length of sliding time window). Second, a subset of *n* nodes is selected (for instance the upper triangle of the matrix excluding the diagonal on Fig 11). Third, a *T* × *T* matrix is calculated where each (*t*_1_, *t*_2_) couple reports the correlation among the two *n*-values vectors indexed at time *t*_1_ and *t*_2_. This *T* × *T* matrix is referred to as the Functional Connectivity Dynamics matrix (FCD).

### Models

The connectome has a space-time structure (structural links, length of tracts). The time delays via signal transmission (equalling tract length divided by transmission speed) are ignored, because we consider only fixed-point models at network nodes (see for instance [20]). We use the connectome as the coupling in a recurrent network, which offers a natural support for fixed-point multistability similar to the spin-glass model [39] and subsequent studies on auto-associative memories extensively studied in the 80’s [59]. Hopfield’s seminal paper popularized this concept as a key element of associative memory, introducing the tools and ideas of statistical physics to neural networks modeling. Several studies demonstrated that the attractor stability decreases with the number of items stored in the network [40] and the maximal number of possible memories linearly grows with the number of neurons [60], or with the number of synapses in the large dilution limit [61]. The extension of the initial model to graded neuronal responses [18] qualitatively provides similar properties [62].

The original Hopfield model [59] is a dynamical system with discrete time steps and composed of multiple nodes. The interactions between the nodes rely on a connectivity matrix built from a pre-existing set of prototypes. The update, inspired by the spin-glass [39] model (used to describe magnetic properties of dilute alloys), is based on a random scan of nodes. The existence of a fixed point dynamics is guaranteed by a Lyapunov function. Hopfield gave conditions, under which every prototype is an attractor of the multistable network dynamics [59]. When the initial conditions are close enough to one of the prototypes, the dynamical system relaxes to the corresponding attractor. When the network dynamics relaxes into an attractor, then this process is interpreted as a retrieval of a stored memory.

Various extensions of the initial model have been proposed. We employ the “graded-response” network model [18], which has a similar Lyapunov function and offers a more intuitive physiological interpretation. This time-continuous version is similar to the Wilson-Cowan model [63] and belongs to the family of “neural mass” models where the activity of a node is interpreted as the collective activity of a set of neurons, typically their average firing rate. The dynamical system is then described by its *N* state variables *x*_1_, …, *x*_*N*_. The connections between the nodes are described by a matrix *W*, whose entries are normalized, i.e. ||*W*|| = 1. The matrix elements are taken from the connectome, where *W* = *C*/||*C*|| and *C* is the original connectome. Each node activity is determined by the weighted sum across the activity of all input nodes and experiences a linear decay (see Eq (1)), where *τ*_*x*_ defines the time scale of the dynamics. The transfer function is a monotonic sigmoid with activation threshold *θ*_*i*_ bounded between 0 (no output) and 1 (strong output) (see Eq (2)).

$$\begin{array}{ccc} \tau_{x}\frac{dx_{i}}{dt} & = & -x_{i}+\sum_{j=1}^{N}W_{i,j}A_{j} \end{array}$$ (1)

$$\begin{array}{ccc} A_{i} & = & \frac{1}{2}\left(1+\tanh\left(G\left(Px_{i}-\theta_{i}\right)\right)\right) \end{array}$$ (2)

where *x*_*i*_ is the node potential, *A*_*i*_ the node output, *W* the connectivity matrix, *θ*_*i*_ the threshold, *P* the scaling factor, *G* the gain and *τ*_*x*_ the time constant.

The activation threshold is an essential component of the network model. Classical Hopfield models use a different threshold on every node (Static and Local) (Eq (3)). Note that here only the model with *P* = 1 exactly corresponds to the original Hopfield model, in which case the dynamical system is symmetric around *A*_*i*_ = 0.5. Each node has an equal probability to be active or inactive (provided its local weights sum is different than 0). For *P* ≠ 1, the activity is biased, either toward a higher proportion of nodes active for *P* > 1, or a lesser proportion for *P* < 1.

We consider here several variants of the model, that is the case of static global threshold (Static and Global—SG) (Eq (4)), and the case of dynamic global threshold (Dynamic and Global—DG) (Eq (5)).

$$\begin{array}{c} \theta_{i}=\frac{1}{2}\sum_{j=1}^{N}W_{i,j}\;\;\;\;\text{SL} \end{array}$$ (3)

$$\begin{array}{c} \theta =\frac{1}{2N}\sum_{i=1}^{N}\sum_{j=1}^{N}W_{i,j}\;\;\;\;\text{SG} \end{array}$$ (4)

$$\begin{array}{c} \tau_{\theta }\frac{d\theta }{dt}=-\theta +\sum_{i=1}A_{i}/N\;\;\;\;\text{DG} \end{array}$$ (5)

The SG model (Eq (4)) is a natural simplification of the original Hopfield model. Replacing the local thresholds by a single one has strong implications, discarding the symmetry around the central 0.5 state. Each node continues to be active when a large enough proportion of its inputs is active. In this setting, as the weight averages are not balanced between the nodes, some nodes with greater (resp. lower)-than-average weight have a greater (resp. lower) probability to be active than the others. In order to keep similar parametric ranges as in the first model, the global threshold is calculated as the average over the local thresholds of (Eq (3)).

In the DG model, (Eq (5)), the threshold changes over time as a function of the node activities. The general idea is to decrease the threshold when the nodes’ activity is too low, and to increase the threshold when the activity is too high. As such, the threshold participates dynamically in the process and exerts a regulatory influence on the dynamics, i.e. controlling the average level of activity (in the spirit of e.g. [60]). The dynamic threshold could, for instance, be realized via a local population of inhibitory neurons (with linear response). In this case, the scaling parameter *P* would represent the Excitatory/Inhibitory (*E*/*I*) ratio. *P* < 1 means that the inhibition dominates the excitation, and *P* > 1 means the opposite. Following Eq (3), the dynamic threshold is calculated as the average over all nodes’ activity, representing a spatio-temporal average, i.e. the average proportion of active nodes in the system. From a biological standpoint, we interprete it as a global inhibitory node (with linear response) feeding back the average activity toward every excitatory node of the system (see Fig 1).

We implement a stochastic generalization of the above networks via the following equations:

$$\begin{array}{c} \tau_{x}\frac{dx_{i}}{dt}=-x_{i}+\sum_{j=1}W_{i,j}A_{j}+\sigma_{x}\eta_{i}\left(t\right) \end{array}$$ (6)

and

$$\begin{array}{c} \tau_{\theta }\frac{d\theta_{i}}{dt}=-\theta_{i}+\theta_{i}^{0}+\sigma_{\theta }\xi_{i}\left(t\right)\;\;\;\;\text{SL} \end{array}$$ (7)

$$\begin{array}{c} \tau_{\theta }\frac{d\theta }{dt}=-\theta +\theta^{0}+\sigma_{\theta }\xi \left(t\right)\;\;\;\;\text{SG} \end{array}$$ (8)

$$\begin{array}{c} \tau_{\theta }\frac{d\theta }{dt}=-\theta +\sum_{i=1}A_{i}/N+\sigma_{\theta }\xi \left(t\right)\;\;\;\;\text{DG} \end{array}$$ (9)

where white Gaussian noise terms *η*_*i*_ and *ξ*_*i*_ are added linearly to the evolution equations with corresponding diffusion strengths *σ*_*x*_ and *σ*_*θ*_, respectively.

### Numerical simulations

The deterministic computer simulations have been performed using the Euler discretization scheme with discrete steps of 0.1 ms. When not pointed out otherwise, the initial state is a random binary vector **A**_0_, whose proportion of zeros and ones is set according to a density factor *f*_0_. The initial conditions are set randomly according to a binomial draw with an expectation varying from 0 to 1 according to *f*_0_. After initialization, the activity of the nodes is recurrently transmitted to the other nodes through the structural connectivity matrix. In the noiseless case, the dynamics is expected to relax on a stable attractor in short time. Let ***A*** ^*k*^(0) be the *k* ^th^ vector of initial conditions and ***A*** ^*k*^(*t*) the corresponding vector of activity at time *t*. The average activity at time *t* (see Figs 2a, 10a, 10c and 10e) is defined as ${\bar{A}}^{k}\left(t\right)=\frac{1}{N}\sum_{i=1}^{N}A_{i}^{k}\left(t\right)$. The state variable time constant is set to a fixed value *τ*_*x*_ = 10 ms. The threshold time constant *τ*_*θ*_, mostly representing inhibitory influences, is considered slower and varied from 10 ms (identical time scales) to 80 ms (time scales separation). The scaling factor *P* is a pivotal parameter interpreted as the system excitatory/inhibitory balance. Depending on the model, *P* varies from 0 (no excitation) to 20 (strong excitation). The noise strengths *σ*_*x*_ and *σ*_*θ*_ are set within the interval [0, 1].

### Random sampling-based attractor search

In analogy with Hopfield’s neural network interpretation, we assume several attractors to be “stored” via the coupling matrix, here given by the connectome. We use the following reverse approach: given a particular connectivity matrix, we try to infer the set of prototypes embedded in it. To do so, we sample the initial conditions and randomly initialize the system with binary activation patterns (namely active or inactive node). Since the number of initial configurations grows exponentially with the size, we only take a sample from a subset of possible initial states. Importantly, we consider different initial densities in order to better sample the state space, varying from 0.02 to 0.98 with 0.03 steps (here 33 densities distributed linearly on the ]0,1[ interval—excluding 0 corresponding to a trivial solution for the continuous Hopfield model). In the DG model, the threshold time constant is set to the same value as the potential time constant. Then, for each density, we let *n* different initial conditions relax on their attractor. For each value of the parameter space to study, 33 × *n* random initialization are thus processed. In the particular case where the initial density is parametrized, *n* = 3,300 initializations are used on a given density parameters. The dynamics is stopped when the system reaches its equilibrium $\frac{{\langle \bar{x}\left(t\right)\rangle }_{T}\;-\bar{x}\left(t\right)}{\bar{x}\left(t\right)}<\varepsilon$ or after 1 second if the equilibrium condition is not reached, where $\bar{x}\left(t\right)$ is the average potential over the nodes (at time *t*), ${\langle \bar{x}\left(t\right)\rangle }_{T}$ is the temporal average of $\bar{x}\left(t\right)$ on the [*t* − *T*, *t*] temporal interval (*T* = 100 ms), and *ε* a (small) constant (10^−6^).

Any final pattern of activity satisfying a double dissimilarity condition is saved, otherwise the cardinality of the better matching attractor set is incremented. The double dissimilarity condition considers both a Pearson correlation *and* a Euclidean similarity lower than 0.9. The goal of this double dissimilarity condition is to discard the low-density patterns, possibly having a low correlation but a high Euclidian similarity, and the high density patterns possibly having a lower Euclidean similarity but being strongly correlated. A final set of *m* ≤ *n* attractors is obtained, each attractor being associated with its cardinality, representing the “width” of its attraction basin.

$$\begin{array}{ccc} {\text{sim}}_{\text{cor}}\left(\text{x},\text{y}\right) & = & \frac{\sum_{i}\left(x_{i}-\bar{x}\right)\left(y_{i}-\bar{y}\right)}{{\left(\text{var}(\text{x})\text{var}(\text{y})\right)}^{1/2}}\\ {\text{sim}}_{\text{eucl}}\left(\text{x},\text{y}\right) & = & \frac{1}{1+{\left(\sum_{i}{\left(x_{i}-y_{i}\right)}^{2}\right)}^{1/2}} \end{array}$$

### Clustering algorithm

For large attractor sets we use clustering algorithms (see Algorithm 1) to identify classes of attractors with spatial similarity. The clustering algorithm operates on binary patterns sets. A binary pattern is composed of active nodes (that is activity > 0.5) and inactive nodes (activity < 0.5). Each binary pattern defines a set of active nodes *A* (and a complementary set of inactive nodes $\bar{A}$). For the purpose of extracting large-scale structural invariants, we use a specific “inclusion match” metric that indicates which proportion of a binary pattern *A* is included in a binary pattern *B*:

$$\begin{array}{c} \text{incl}\left(A,B\right)=\{\begin{array}{cc} \frac{\left|A\cap B\right|}{\left|A\right|} & \;\text{if}\;|A|>0\\ 0 & \;\text{if}\;|A|=0 \end{array} \end{array}$$

Under this metric, patterns of variable size may share elements and are considered similar. For instance, if all the elements of the pattern *A* are included in the pattern *B*, the inclusion match is equal to 1. Because the metric is non-symmetric, we employ the following modification

$$\begin{array}{c} {\text{sim}}_{\text{incl}}\left(A,B\right)=\max\left\{\text{incl}\left(A,B\right),\text{incl}\left(B,A\right)\right\}. \end{array}$$

**Algorithm 1** Clustering algorithm

1: parameter: *k*

2: initialize *n* sets:

*S* ← {{**x**_1_}, …, {**x**_*n*_}}

3: sim_test_ ← 1

4: **while** sim_test_ > *k* **do**

5:  $E,F\leftarrow \underset{(E^{\prime },F^{\prime })\in S\times S}{\text{argmax}}{\text{sim}}_{\text{ref}}\left(E^{\prime },F^{\prime }\right)$

6:  sim_test_ ← sim_ref_(*E*, *F*)

7:  remove *E* and *F* from *S*

8:  *G* ← *E* ∪ *F*

9:  Add *G* to *S*

10: **end while**

The similarity between two clusters *E* and *F* is the similarity between the reference patterns ***μ***_*E*_ and ***μ***_*F*_ of the two sets, i.e. sim_ref_(*E*, *F*) = sim_incl_(***μ***_*E*_, ***μ***_*F*_). The reference patterns can be calculated in different ways (e.g. the average pattern of the cluster, etc.).

In the “double pass” clustering case, the algorithm 1 is applied twice to the same set of patterns. In the first pass, the reference patterns of the clusters are the patterns with the highest “inclusion score”, where the inclusion score is the sum of the inclusion matching with all the other patterns of the cluster. In the second pass, the reference patterns are the average binary patterns, gathering the nodes that are active in more than 50% of the patterns. The first pass results in a set of clusters whose constituents have a variable density, but share an essential “core” activity that is present in all the patterns of a cluster. The second pass is a smoothing pass that gathers together the clusters being similar on average (where the final average patterns are then possibly composed of several “cores”).

### Comparison with rs-fMRI patterns

Consider each rs-fMRI pattern **x** as a set of 250 active nodes in a total of 1,000 nodes (the 250 most active nodes in a cluster of observation vectors). Consider each simulation-based pattern as a set **y** of 100 active nodes in a total of 1,000 nodes (the 100 most active nodes in a cluster of attractors). To test independence, we compare the match between **x** and **y** against a random set of 100 nodes **z**. If **z** is drawn independently from **x**, the expected match $E(\frac{\left|\text{x}\cap \text{z}\right|}{\left|\text{z}\right|})$ is 0.25 with variance $\frac{0.25(1-0.25)}{100}$. Note $m=\frac{\left|\text{x}\cap \text{y}\right|}{\left|\text{y}\right|}$ is the match between **x** and **y**. Then, under the Normal approximation, the Student t-value is $\frac{\left(m-0.25\right)\times \sqrt{100}}{\sqrt{0.25(1-0.25)}}$, where *t* > 3 (*m* > 0.37) denotes less than 0.1% chance for **x** and **y** to be independent.

### BOLD signal reconstruction

Simulated network data are downsampled with 100 Hz (average values over 10 ms windows). Then the data are convolved with a Balloon/Windkessel kernel [56–58]:

$$\begin{array}{c} H\left(t\right)=\exp(\frac{-0.5\;t}{\tau_{s}})\;\frac{\sin(t\;\sqrt{\frac{1}{\tau_{f}}-\frac{1}{4\;\tau_{s}\;T}})}{\sqrt{\frac{1}{\tau_{f}}-\frac{1}{4\;\tau_{s}\;T}}} \end{array}$$ (10)

with *T* = 10, *τ*_*s*_ = 0.8 s, *τ*_*f*_ = 0.4 s. The resulting signal is finally downsampled at 0.5 Hz and detrended (by suppressing the time-averaged value).

### Potential function

Our modeling approach allows deriving analytically a potential function, from which many dynamic and stochastic properties of the network can be derived in a simple manner. We begin our discussion with the formulation of the Fokker-Planck equation and its solutions. As the resting state dynamics evolves, it traces out a trajectory in a *M*-dimensional state space. The *M*-dimensional state vector **q**(*t*) = (…*q*_i_(*t*)…) obeys the Langevin equation $\dot{\text{q}}\left(t\right)=\text{K}\left(\text{q}\left(t\right)\right)+\text{F}\left(t\right)$ where the rate of change of the state vector (time derivative on the left) depends on its deterministic influences **K** = (…*K*_*i*_(*t*)…) that are non-linearly dependent on its current state and stochastic forces **F** = (…*F*_*i*_(*t*)…). Here we consider only *δ*-correlated fluctuating forces **F**, i.e. <*F*_*i*_(*t*)*F*_*j*_(*t*′)> = *Q*_*i*, *j*_ *δ*(*t* − *t*′). The probability density function *f*(**q**, *t*) defines the distribution realizations of trajectories in **q**-space and its dynamics is determined by the Fokker-Planck equation.

$$\begin{array}{c} \dot{f}=-\nabla_{\text{q}}\left\{\text{K}f\right\}-\frac{1}{2}\sum_{k,l}Q_{k,l}\frac{\partial^{2}f}{\partial q_{k}\partial q_{l}} \end{array}$$ (11)

We can rewrite the Fokker-Planck equation as a continuity equation by means of the abbreviation $j_{k}=\left(K_{k}f-\frac{1}{2}\sum_{l}Q_{k,l}\frac{\partial f}{\partial_{q_{l}}}\right)$ and obtain $\dot{f}=-\nabla_{q}\circ \text{j}$, where the temporal change of the probability density *f*(**q**) is equal to the negative divergence of the probability current **j** = (…*j*_*k*_…). For $\dot{f}=0$, the stationary solution of the Fokker-Planck equation is time-independent. The stationary solution does not imply zero current, **j** = **0**, because closed probability flows may persist. The deterministic components may be expressed via a gradient dynamics as in the case of the SL and SG models (**q** = **x**, *M* = *N*), then this allows us to determine an explicit stationary solution of the Fokker-Planck equation. In particular, when $K_{k}=-\frac{\partial V(x)}{\partial x_{k}}$ and the diffusion coefficients obey the condition *Q*_*k*,*l*_ = *δ*_*k*,*l*_ *Q*, then the time independent probability density function reads:

$$\begin{array}{c} f\left(\text{x}\right)=n\exp(-2V(\text{x})/Q) \end{array}$$ (12)

where *n* is the normalization coefficient, satisfying the natural boundary conditions that *f*(**x**) vanishes for |**x**| → ∞. For our large-scale network, the potential function *V*(**x**) reads:

$$\begin{array}{c} V\left(\text{x}\right)=\frac{1}{2}\sum_{i}[x_{i}^{2}-x_{i}\sum_{j}W_{i,j}-x_{i}\sum_{j\neq i}W_{i,j}\text{tanh}\left(G\left(Px_{j}-\theta_{j}\right)\right)-W_{i,i}\frac{1}{GP}\text{ln}\;\text{cosh}\left(G\left(Px_{i}-\theta_{i}\right)\right)] \end{array}$$ (13)

### Stationary distribution

The minima of the potential function *V*(**x**) determine the set of potential states that the large-scale brain network can occupy and thus defines its dynamical repertoire. The stationary time independent solution of the probability density allows characterizing the most likely paths to be taken in state space by identifying regions of high probability. This analytical approach turns out to be a major advantage as opposed to computational approaches, which would require time-consuming simulations of many realizations of trajectories. The following crucial distinction needs to be made here: what is commonly referred to as non-stationary brain dynamics at rest does generally not refer to a non-stationary dynamics of the probability density, but rather to the evolution of the trajectory in state space occupying certain subspaces for a finite time, followed by a rapid switch to occupy another subspace and dwell there for another characteristic time. The realized probability density is stationary and described by Eq (12). Time-dependent solutions of the Fokker-Planck equation cannot be expressed analytically in general terms, but formulated at least locally around a given brain state (minimum of *V*(**x**)), which allows the explicit computation of the time dependent moments such as the time-dependent mean around a brain state or its two-time correlation function [64].

For a large excitability *G*, the sigmoidal saturation function in our network model approximates a Heavyside function which approximates a reduced Ising-spin attractor model, which allows further analytical investigation as performed by Deco et al [42]. The model is then a network of stochastic binary units (“spins”), where each unit *s*_*i*_ takes output value *A*_*i*_ = 1 with probability *f*_*i*_ and value *A*_*i*_ = 0 with probability 1 − *f*_*i*_. For symmetric connectivity, the Boltzmann-Gibbs distribution giving the probability of finding the network in a specific state **A** ^*α*^ can be expressed analytically by

$$\begin{array}{c} p^{\alpha }=\frac{e^{-\beta E^{\alpha }}}{Z}. \end{array}$$ (14)

where *Z* is the partition function defined by

$$\begin{array}{c} Z=\sum_{\alpha }e^{-\beta E^{\alpha }} \end{array}$$ (15)

and *E* ^*α*^ is the energy function

$$\begin{array}{c} E^{\alpha }=-\frac{1}{2}\sum_{i,j}W_{i,j}A_{i}^{\alpha }A_{j}^{\alpha }-\sum_{i}\theta_{i}A_{i}^{\alpha } \end{array}$$ (16)

The probability *p* ^*α*^ gives the probability of finding the configuration **A** ^*α*^. Therefore, in order to describe the attractor landscape of the spin network, we can characterize the existence and probability of each possible attractor (here corresponding to a specific configuration **A** ^*α*^) by the entropy of the system, which can be derived analytically, yielding:

$$\begin{array}{c} H=-\sum_{\alpha }p^{\alpha }\log p^{\alpha }=\frac{\sum_{\alpha }\beta E^{\alpha }e^{-\beta E^{\alpha }}}{Z}+\log Z \end{array}$$ (17)

Deco et al. [42] computed explicitly different types of structural networks and investigated how the entropy of the Ising-spin network evolves as a function of connectivity. The higher the entropy is, the larger is the number of “ghost” attractors that efficiently structure the fluctuations of the system at the edge of the bifurcation. The empirical entropies (shown in Figs 3 and 5) rely on the cardinality of the different sets of attractors obtained after sampling the initial condition space. Namely, for each attractor *i*, ${\tilde{p}}_{i}=\frac{n_{i}}{n}$ where *n*_*i*_ is the cardinal of the *i* ^th^ attractor set and *n* is the number of samples. Then, ∀*i*:

$$\begin{array}{c} \tilde{H}=-\sum_{i}{\tilde{p}}_{i}\log{\tilde{p}}_{i} \end{array}$$ (18)

## References

[1] Biswal B, Yetkin FZ, Haughton VM, Hyde JS. Functional connectivity in the motor cortex of resting human brain using echo-planar MRI. Magnetic resonance in medicine: official journal of the Society of Magnetic Resonance in Medicine / Society of Magnetic Resonance in Medicine. 1995;34:537–541. 10.1002/mrm.1910340409 8524021

[2] Beckmann CF, DeLuca M, Devlin JT, Smith SM. Investigations into resting-state connectivity using independent component analysis. Philosophical transactions of the Royal Society of London Series B, Biological sciences. 2005;360:1001–13. Available from: http://www.ncbi.nlm.nih.gov/pubmed/16087444 10.1098/rstb.2005.1634 16087444 PMC1854918

[3] Fox MD, Corbetta M, Snyder AZ, Vincent JL, Raichle ME. Spontaneous neuronal activity distinguishes human dorsal and ventral attention systems. Proceedings of the National Academy of Sciences of the United States of America. 2006;103:10046–51. Available from: http://www.pubmedcentral.nih.gov/articlerender.fcgi?artid=1480402&tool=pmcentrez&rendertype=abstract 10.1073/pnas.0604187103 16788060 PMC1480402

[4] Damoiseaux JS, Rombouts SaRB, Barkhof F, Scheltens P, Stam CJ, Smith SM, et al Consistent resting-state networks across healthy subjects. Proceedings of the National Academy of Sciences of the United States of America. 2006;103:13848–13853. 10.1073/pnas.0601417103 16945915 PMC1564249

[5] Seeley WW, Menon V, Schatzberg AF, Keller J, Glover GH, Kenna H, et al Dissociable intrinsic connectivity networks for salience processing and executive control. The Journal of neuroscience: the official journal of the Society for Neuroscience. 2007;27:2349–56. Available from: http://www.pubmedcentral.nih.gov/articlerender.fcgi?artid=2680293&tool=pmcentrez&rendertype=abstract 10.1523/JNEUROSCI.5587-06.2007 17329432 PMC2680293

[6] Mantini D, Perrucci MG, Del Gratta C, Romani GL, Corbetta M. Electrophysiological signatures of resting state networks in the human brain. Proceedings of the National Academy of Sciences of the United States of America. 2007;104:13170–13175. 10.1073/pnas.0700668104 17670949 PMC1941820

[7] Smith SM, Fox PT, Miller KL, Glahn DC, Fox PM, Mackay CE, et al Correspondence of the brain’s functional architecture during activation and rest. Proceedings of the National Academy of Sciences of the United States of America. 2009;106:13040–5. Available from: http://www.ncbi.nlm.nih.gov/pubmed/19620724 10.1073/pnas.0905267106 19620724 PMC2722273

[8] Meunier D, Lambiotte R, Fornito A, Ersche KD, Bullmore ET. Hierarchical modularity in human brain functional networks. Frontiers in neuroinformatics. 2009;3:37 10.3389/neuro.11.037.2009 19949480 PMC2784301

[9] Raichle ME. The restless brain. Brain Connectivity. 2011;1(1):3–12. 10.1089/brain.2011.0019 22432951 PMC3621343

[10] Power JD, Cohen AL, Nelson SM, Wig GS, Barnes KA, Church JA, et al Functional network organization of the human brain. Neuron. 2011;72:665–78. Available from: http://www.pubmedcentral.nih.gov/articlerender.fcgi?artid=3222858&tool=pmcentrez&rendertype=abstract 10.1016/j.neuron.2011.09.006 22099467 PMC3222858

[11] Wu GR, Stramaglia S, Chen H, Liao W, Marinazzo D. Mapping the voxel-wise effective connectome in resting state FMRI. PLoS One. 2013;8:e73670 Available from: http://www.ncbi.nlm.nih.gov/pubmed/24069220 10.1371/journal.pone.0073670 24069220 PMC3771991

[12] Allen Ea, Damaraju E, Plis SM, Erhardt EB, Eichele T, Calhoun VD. Tracking whole-brain connectivity dynamics in the resting state. Cerebral Cortex. 2014;24:663–676. 10.1093/cercor/bhs352 23146964 PMC3920766

[13] Hansen ECA, Battaglia D, Spiegler A, Deco G, Jirsa VK. Functional Connectivity Dynamics: Modeling the switching behavior of the resting state. NeuroImage. 2014;105:525–535. Available from: http://www.sciencedirect.com/science/article/pii/S1053811914009033 10.1016/j.neuroimage.2014.11.001 25462790

[14] Ghosh A, Rho Y, McIntosh aR, Kötter R, Jirsa VK. Noise during rest enables the exploration of the brain’s dynamic repertoire. PLoS computational biology. 2008;4:e1000196 Available from: http://www.ncbi.nlm.nih.gov/pubmed/18846206 10.1371/journal.pcbi.1000196 18846206 PMC2551736

[15] McIntosh aR, Kovacevic N, Lippe S, Garrett D, Grady C, Jirsa V. The development of a noisy brain. Archives Italiennes de Biologie. 2010;148:323–337. 21175017

[16] Nakagawa TT, Jirsa VK, Spiegler A, McIntosh AR, Deco G. Bottom up modeling of the connectome: Linking structure and function in the resting brain and their changes in aging. NeuroImage. 2013;80:318–329. Available from: 10.1016/j.neuroimage.2013.04.055 10.1016/j.neuroimage.2013.04.055 23629050

[17] Sleimen-Malkoun R, Perdikis D, Müller V, Blanc Jl, Huys R, Temprado Jj, et al. Brain Dynamics of Aging: Multiscale Variability of EEG Signals at Rest and during an Auditory Oddball Task. eNeuro. 2015;2. Available from: http://eneuro.org/content/2/3/ENEURO.0067-14.2015 10.1523/ENEURO.0067-14.2015 PMC4586928 26464983

[18] Hopfield JJ. Neurons with graded response have collective computational properties like those of two-state neurons. Proceedings of the National Academy of Sciences of the United States of America. 1984;81:3088–3092. 10.1073/pnas.81.10.3088 6587342 PMC345226

[19] Deco G, Jirsa V, McIntosh aR, Sporns O, Kötter R. Key role of coupling, delay, and noise in resting brain fluctuations. Proceedings of the National Academy of Sciences of the United States of America. 2009;106:10302–7. Available from: http://www.pubmedcentral.nih.gov/articlerender.fcgi?artid=2690605&tool=pmcentrez&rendertype=abstract 10.1073/pnas.0901831106 19497858 PMC2690605

[20] Deco G, Jirsa VK. Ongoing cortical activity at rest: Criticality, multistability, and ghost attractors. J Neurosci. 2012;32:3366–3375. Available from: http://www.ncbi.nlm.nih.gov/pubmed/22399758 10.1523/JNEUROSCI.2523-11.2012 22399758 PMC6621046

[21] Moran RJ, Kiebel SJ, Stephan KE, Reilly RB, Daunizeau J, Friston KJ. A neural mass model of spectral responses in electrophysiology. NeuroImage. 2007;37:706–20. Available from: http://www.ncbi.nlm.nih.gov/pubmed/17632015 10.1016/j.neuroimage.2007.05.032 17632015 PMC2644418

[22] Stefanescu Ra, Jirsa VK. A low dimensional description of globally coupled heterogeneous neural networks of excitatory and inhibitory neurons. PLoS computational biology. 2008;4:e1000219 Available from: http://www.pubmedcentral.nih.gov/articlerender.fcgi?artid=2574034&tool=pmcentrez&rendertype=abstract 10.1371/journal.pcbi.1000219 19008942 PMC2574034

[23] Moran RJ, Stephan KE, Seidenbecher T, Pape HC, Dolan RJ, Friston KJ. Dynamic causal models of steady-state responses. NeuroImage. 2009;44:796–811. Available from: http://www.ncbi.nlm.nih.gov/pubmed/19000769 10.1016/j.neuroimage.2008.09.048 19000769 PMC2644453

[24] Jirsa VK, Stefanescu Ra. Neural Population Modes Capture Biologically Realistic Large Scale Network Dynamics. Bulletin of Mathematical Biology. 2011;73:325–343. 10.1007/s11538-010-9573-9 20821061

[25] Stefanescu Ra, Jirsa VK. Reduced representations of heterogeneous mixed neural networks with synaptic coupling. Physical Review E—Statistical, Nonlinear, and Soft Matter Physics. 2011;83:1–12. 10.1103/PhysRevE.83.026204 21405893

[26] Jirsa VK. Dispersion and time delay effects in synchronized spike-burst networks. Cognitive neurodynamics. 2008;2:29–38. Available from: http://www.pubmedcentral.nih.gov/articlerender.fcgi?artid=2289254&tool=pmcentrez&rendertype=abstract 10.1007/s11571-007-9030-0 19003471 PMC2289254

[27] Deco G, Jirsa VK, McIntosh AR. Emerging concepts for the dynamical organization of resting-state activity in the brain. Nature reviews Neuroscience. 2011;12:43–56. 10.1038/nrn2961 21170073

[28] Moran RJ, Stephan KE, Dolan RJ, Friston KJ. Consistent spectral predictors for dynamic causal models of steady-state responses. NeuroImage. 2011;55:1694–1708. Available from: 10.1016/j.neuroimage.2011.01.012 10.1016/j.neuroimage.2011.01.012 21238593 PMC3093618

[29] Ritter P, Schirner M, McIntosh AR, Jirsa VK. The virtual brain integrates computational modeling and multimodal neuroimaging. Brain connectivity. 2013;3:121–45. Available from: http://online.liebertpub.com/doi/abs/10.1089/brain.2012.0120 10.1089/brain.2012.0120 23442172 PMC3696923

[30] Feng J, Jirsa VK, Ding M. Synchronization in networks with random interactions: Theory and applications. Chaos. 2006;16:15109 Available from: http://www.ncbi.nlm.nih.gov/pubmed/16599775 10.1063/1.2180690 16599775

[31] Sanz Leon P, Knock Sa, Woodman MM, Domide L, Mersmann J, McIntosh AR, et al The Virtual Brain: a simulator of primate brain network dynamics. Frontiers in neuroinformatics. 2013;7:10 Available from: http://www.pubmedcentral.nih.gov/articlerender.fcgi?artid=3678125&tool=pmcentrez&rendertype=abstract 10.3389/fninf.2013.00010 23781198 PMC3678125

[32] Spiegler A, Jirsa VK. Systematic approximations of neural fields through networks of neural masses in the virtual brain. NeuroImage. 2013;83:704–25. Available from: http://www.ncbi.nlm.nih.gov/pubmed/23774395 10.1016/j.neuroimage.2013.06.018 23774395

[33] Woodman MM, Pezard L, Domide L, Knock Sa, Sanz-Leon P, Mersmann J, et al Integrating neuroinformatics tools in The Virtual Brain. Frontiers in Neuroinformatics. 2014;8:36 Available from: http://www.pubmedcentral.nih.gov/articlerender.fcgi?artid=4001068&tool=pmcentrez&rendertype=abstract 10.3389/fninf.2014.00036 24795617 PMC4001068

[34] Sanz-Leon P, Knock Sa, Spiegler A, Jirsa V. Mathematical framework for large-scale brain network modelling in The Virtual Brain. Neuroimage. 2015;111:385–430. Available from: http://www.sciencedirect.com/science/article/pii/S1053811915000051 10.1016/j.neuroimage.2015.01.002 25592995

[35] Zandt BJ, ten Haken B, van Putten MJAM, Dahlem MA. How does spreading depression spread? Physiology and modeling. Reviews in the Neurosciences. 2015;26:183–198. Available from: <GotoISI>://WOS:000351924800005 25719306 10.1515/revneuro-2014-0069

[36] Hübel N, Dahlem Ma. Dynamics from seconds to hours in hodgkin-huxley model with time-dependent ion concentrations and buffer reservoirs. PLoS computational biology. 2014;10:e1003941 Available from: http://www.pubmedcentral.nih.gov/articlerender.fcgi?artid=4256015&tool=pmcentrez&rendertype=abstract 10.1371/journal.pcbi.1003941 25474648 PMC4256015

[37] El Houssaini K, Ivanov AI, Bernard C, Jirsa VK. Seizures, refractory status epilepticus, and depolarization block as endogenous brain activities. Physical Review E. 2015;91(1):010701 10.1103/PhysRevE.91.010701 25679555

[38] Hagmann P, Cammoun L, Gigandet X, Meuli R, Honey CJ, Wedeen VJ, et al Mapping the structural core of human cerebral cortex. PLoS biology. 2008;6:e159 Available from: http://www.ncbi.nlm.nih.gov/pubmed/18597554 10.1371/journal.pbio.0060159 18597554 PMC2443193

[39] Sherrington D, Kirkpatrick S. Solvable Model of a Spin-Glass. Physical Review Letters. 1975;35:1792–1796. Available from: http://link.aps.org/doi/10.1103/PhysRevLett.35.1792 10.1103/PhysRevLett.35.1792

[40] Amit D, Gutfreund H, Sompolinsky H. Spin-glass models of neural networks; 1985. 10.1103/physreva.32.1007 9896156

[41] Amit DJ, Gutfreund H, Sompolinsky H. Statistical mechanics of neural networks near saturation. Annals of Physics. 1987;173:30–67. 10.1016/0003-4916(87)90092-3

[42] Deco G, Senden M, Jirsa V. How anatomy shapes dynamics: a semi-analytical study of the brain at rest by a simple spin model. Frontiers in Computational Neuroscience. 2012;6:1–7. 10.3389/fncom.2012.00068 23024632 PMC3447303

[43] Van Essen D, Smith S, Barch D, Behrens T, Yacoub E, Ugurbil K. The WU-Minn Human Connectome Project: An overview. Neuroimage. 2013;80:62–79. 10.1016/j.neuroimage.2013.05.041 23684880 PMC3724347

[44] Proix T. Surface and Connectivity Reconstruction: Imaging Pipeline & ToolS—SCRIPTS; 2014. [Online; 25-Mars-2014]. https://github.com/timpx/scripts

[45] Honey CJ, Honey CJ, Sporns O, Sporns O, Cammoun L, Cammoun L, et al Predicting human resting-state functional connectivity from structural connectivity. Proceedings of the National Academy of Sciences of the United States of America. 2009;106:2035–40. Available from: http://www.ncbi.nlm.nih.gov/pubmed/19188601 10.1073/pnas.0811168106 19188601 PMC2634800

[46] Utevsky AV, Smith DV, Huettel Sa. Precuneus is a functional core of the default-mode network. The Journal of neuroscience: the official journal of the Society for Neuroscience. 2014;34:932–40. Available from: http://www.pubmedcentral.nih.gov/articlerender.fcgi?artid=3891968&tool=pmcentrez&rendertype=abstract 10.1523/JNEUROSCI.4227-13.2014 24431451 PMC3891968

[47] Messé A, Rudrauf D, Benali H, Marrelec G. Relating structure and function in the human brain: relative contributions of anatomy, stationary dynamics, and non-stationarities. PLoS computational biology. 2014;10:e1003530 Available from: http://www.pubmedcentral.nih.gov/articlerender.fcgi?artid=3961181&tool=pmcentrez&rendertype=abstract 10.1371/journal.pcbi.1003530 24651524 PMC3961181

[48] Raichle ME, MacLeod aM, Snyder aZ, Powers WJ, Gusnard Da, Shulman GL. A default mode of brain function. Proceedings of the National Academy of Sciences of the United States of America. 2001;98:676–682. 10.1073/pnas.98.2.676 11209064 PMC14647

[49] Jirsa VK, Sporns O, Breakspear M, Deco G, Mcintosh aR. Towards the virtual brain: Network modeling of the intact and the damaged brain. Archives Italiennes de Biologie. 2010;148:189–205. 21175008

[50] Deco G, Jirsa VK, McIntosh AR. Resting brains never rest: Computational insights into potential cognitive architectures. Trends in Neurosciences. 2013;36:268–274. Available from: 10.1016/j.tins.2013.03.001 10.1016/j.tins.2013.03.001 23561718

[51] van den Heuvel MP, Stam CJ, Boersma M, Hulshoff Pol HE. Small-world and scale-free organization of voxel-based resting-state functional connectivity in the human brain. NeuroImage. 2008;43:528–39. Available from: http://www.ncbi.nlm.nih.gov/pubmed/18786642 10.1016/j.neuroimage.2008.08.010 18786642

[52] Zalesky A, Fornito A, Harding IH, Cocchi L, Yücel M, Pantelis C, et al Whole-brain anatomical networks: Does the choice of nodes matter? NeuroImage. 2010;50:970–983. Available from: 10.1016/j.neuroimage.2009.12.027 10.1016/j.neuroimage.2009.12.027 20035887

[53] Crick FC, Koch C. What is the function of the claustrum? Philosophical transactions of the Royal Society of London Series B, Biological sciences. 2005;360:1271–1279. 10.1098/rstb.2005.1661 16147522 PMC1569501

[54] Koubeissi MZ, Bartolomei F, Beltagy A, Picard F. Electrical stimulation of a small brain area reversibly disrupts consciousness. Epilepsy and Behavior. 2014;37:32–35. Available from: 10.1016/j.yebeh.2014.05.027 10.1016/j.yebeh.2014.05.027 24967698

[55] Maslov S, Sneppen K. Specificity and stability in topology of protein networks. Science (New York, NY). 2002;296:910–913. 10.1126/science.1065103 11988575

[56] Buxton RB, Frank LR. A model for the coupling between cerebral blood flow and oxygen metabolism during neural stimulation. Journal of Cerebral Blood Flow and Metabolism. 1997;17:64–72. Available from: http://www.nature.com/jcbfm/journal/v17/n1/abs/9590177a.html$\delimiter“026E30F$npapers3://publication/doi/10.1097/00004647-199701000-00009 10.1097/00004647-199701000-00009 8978388

[57] Buxton RB, Wong EC, Frank LR. Dynamics of blood flow and oxygenation changes during brain activation: the balloon model. Magnetic Resonance in Medicine. 1998;39:855–864. Available from: http://www.ncbi.nlm.nih.gov/pubmed/9621908 10.1002/mrm.1910390602 9621908

[58] Friston KJ, Mechelli A, Turner R, Price CJ. Nonlinear responses in fMRI: the Balloon model, Volterra kernels, and other hemodynamics. NeuroImage. 2000;12:466–77. Available from: http://www.ncbi.nlm.nih.gov/pubmed/10988040 10.1006/nimg.2000.0630 10988040

[59] Hopfield JJ. Neural networks and physical systems with emergent collective computational abilities. Proceedings of the National Academy of Sciences of the United States of America. 1982;79:2554–2558. 10.1073/pnas.79.8.2554 6953413 PMC346238

[60] Amit D, Gutfreund H, Sompolinsky H. Information storage in neural networks with low levels of activity. Physical Review A. 1987;35:2293–2303. Available from: http://link.aps.org/doi/10.1103/PhysRevA.35.2293 10.1103/PhysRevA.35.2293 9898407

[61] Tsodyks M, Feigel’man MV. The Enhanced Storage Capacity in Neural Networks with Low Activity Level. Europhysics Letters (EPL). 1988;6:101–105. Available from: http://stacks.iop.org/0295-5075/6/i=2/a=002 10.1209/0295-5075/6/2/002

[62] Kühn R, Bös S, van Hemmen JL. Statistical mechanics for networks of graded-response neurons. Physical Review A. 1991;43(4):2084 10.1103/PhysRevA.43.2084 9905258

[63] Wilson HR, Cowan JD. A mathematical theory of the functional dynamics of cortical and thalamic nervous tissue. Biological Cybernetics. 1973;13:55–80. Available from: http://www.springerlink.com/content/m28540k4769650u4/abstract/ 10.1007/BF00288786 4767470

[64] Haken H. Advanced synergetics. Springer; 1983.

